# Multiple social network influences can generate unexpected environmental outcomes

**DOI:** 10.1038/s41598-021-89143-1

**Published:** 2021-05-07

**Authors:** J. Yletyinen, G. L. W. Perry, P. Stahlmann-Brown, R. Pech, J. M. Tylianakis

**Affiliations:** 1 University of Canterbury, School of Biological Sciences, Private Bag 4800, Christchurch, 8140 New Zealand; 2 Manaaki Whenua-Landcare Research, PO Box 69040, Lincoln, 7640 New Zealand; 3 School of Environment, University of Auckland, Private Bag 92019, Auckland, New Zealand; 4 Manaaki Whenua-Landcare Research, PO Box 10345, Wellington, 6011 New Zealand

**Keywords:** Environmental social sciences, Sustainability, Conservation biology

## Abstract

Understanding the function of social networks can make a critical contribution to achieving desirable environmental outcomes. Social-ecological systems are complex, adaptive systems in which environmental decision makers adapt to a changing social and ecological context. However, it remains unclear how multiple social influences interact with environmental feedbacks to generate environmental outcomes. Based on national-scale survey data and a social-ecological agent-based model in the context of voluntary private land conservation, our results suggest that social influences can operate synergistically or antagonistically, thereby enabling behaviors to spread by two or more mechanisms that amplify each other’s effects. Furthermore, information through social networks may indirectly affect and respond to isolated individuals through environmental change. The interplay of social influences can, therefore, explain the success or failure of conservation outcomes emerging from collective behavior. To understand the capacity of social influence to generate environmental outcomes, social networks must not be seen as ‘closed systems’; rather, the outcomes of environmental interventions depend on feedbacks between the environment and different components of the social system.

## Introduction

Solving environmental problems requires collective effort, including adoption of pro-environmental behaviors^1–3^. A major barrier for individuals to adopt pro-environmental behaviors is being embedded in a social context in which others do not approve of that behavior^1^. Leveraging social influence, basically communicating what “should be done”, may help to overcome this barrier and accelerate the spread of pro-environmental behaviors^1,3,4^. Humans exchange information and knowledge through social interactions, including expressed behaviors, and modify their behaviors and beliefs in response to those of others^1,4,5^. The networked character of social interactions allows behaviors to spread through social networks, potentially creating clusters of people with similar behaviors and views^6–9^. Such behavioral clusters may emerge, for instance, from the tendency for people to form social relationships with like-minded people^10^ (i.e. ‘echo chambers’) into which new ideas cannot easily penetrate. Despite a wealth of work on the importance of social network connections to external actors in environmental management^11^, we know little about how multiple and interacting social influences contribute to the spread of pro-environmental behaviors and emergent environmental outcomes. Studies exploring the effects of social networks on environmental outcomes often focus on one type of social influence at a time, usually an ingroup based on similarity in demographic factors, beliefs, profession etc. (e.g. interactions among fishers^9^)^3,12^. However, individuals experience social influences from people outside the ingroup^13,14^, which may explain unexpected behavioral or environmental outcomes emerging from social groups.

A social-ecological systems (SES) view holds that human behavior constantly adapts to changing conditions and, in so doing, co-evolves with social and environmental contexts^15,16^. In addition to social influence, the environmental outcomes of behaviors are influenced by the biophysical context of decision-making and heterogeneity in each individual’s beliefs and actions^15,17^. In practice, through micro-scale patterns (such as individual beliefs, behaviors and social interactions), humans (as social actors in SES) collectively create and reinforce macro-scale patterns, such as social network structures, social norms, resource abundance and conservation landscapes^15^. These emerging macro-scale patterns, in turn, feedback to shape actors’ micro-scale behaviors^15,16^. Linking social actors’ behaviors to their decision-making context, and investigating their interplay over time as dynamic two-way interactions, is especially important for understanding environmental outcomes emerging from social actors’ collective behaviors^16^. For instance, if social influence leads to the adoption of pro-environmental behaviors, such behavioral changes may not persist in a different social-ecological context. Moreover, the presence of multiple social influences may drive the simultaneous spread of desirable and undesirable social influence among social actors, which may break clusters of behaviors that social networks with one type of social influence can contain^e.g.^^9^. This complex adaptive SES perspective of social influence may help to explain why the environmental outcomes of collective behavior can range from success to failure, and why the outcomes of interventions in social influence are inconsistent.

Here, we investigate the effect of multiple social influences on environmental outcomes in dynamic SESs. In particular, we use simulation modelling to ask whether the success of collective environmental action (voluntary habitat conservation on landscape levels) is influenced by inclusion of multiple social influences in individual (landowner) decision-making. Voluntary conservation of natural and semi-natural habitats in agricultural landscapes epitomises a SES in which social influence strongly influences landowners’ pro-environmental behavior^18–21^. The environmental outcomes emerging from individual actions determine conservation success since a species’ persistence in a landscape is predicted by the composition, abundance and spatial configuration of habitats at the landscape level^22,23^. While social processes influence long-term conservation success^24–26^, the role of dynamic feedbacks between social and ecological outcomes must be better integrated into conservation science to improve our ability to achieve conservation goals^27–31^. Feedbacks underlie the persistence of ecologically or societally undesirable or desirable conservation states^e.g.^^32,33^, and incorporating human behavior into environmental systems research (and vice versa) can reveal a richer diversity of feedbacks than either social or ecological research alone^34^. Identifying interactions between multiple elements or processes^31^ can, for example, inform conservation initiatives that explicitly focus on reinforcing or dampening feedbacks of biodiversity loss^29^.

**Figure 1:**
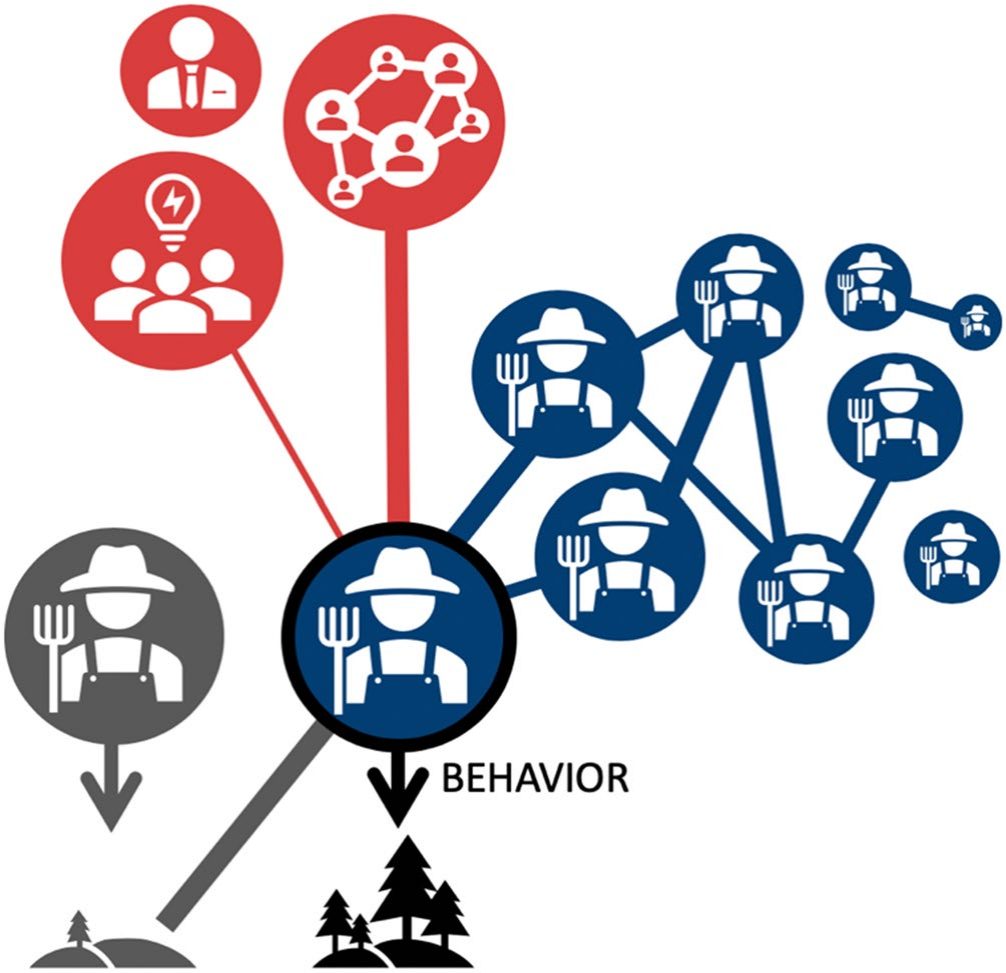
The concept of interacting social influences affecting environmental behavior. In this study, each landowner’s (blue node with black outline) decision about voluntary habitat conservation is affected by their interactions with a network of other landowners (blue nodes), three cross-scale actor groups (red nodes) and spatial knowledge diffusion mediated by change in biophysical context emerging from seeing other landowners’ conservation decisions (exemplified by one grey node). The width of connection between nodes illustrates the level of influence on landowner’s decision-making. The diversity and strength of social influences affecting decision-making vary among landowners. The figure was created using Microsoft PowerPoint version 16.43.

Here, we relax the common assumption in social network analysis that influence and behaviors almost inevitably spread between interacting social actors. Instead, we assume that behavioral decisions are affected by multiple social influences and that environmentally desirable and undesirable behaviors can spread simultaneously^6,35^. Environmental managers, such as landowners, commonly interact with groups of actors with diverse interests^14,20^. Environmental behaviours can, therefore, be influenced by the information and perspectives gained via these interactions and the quality of the interactions, such as level of trust^20^. For example, landowners may contact authorities to gain information about environmental practices that is not available from fellow landowners and then adopt the practice if encouraged to do so by a like-minded landowner^20,36^. In this context, we introduce three types of social influence into landowners’ decisions to voluntarily protect habitat on their own land, based on data collected in a large online survey of rural landowners and land managers (hereafter, 'landowners’) in New Zealand (hereafter, ‘the survey’) (Fig. 1)^37^.

First, *peer influence* captures the frequency and perceived importance of conversations with other landowners about environmental performance on farms (the term “peer” in social networks may take on different meanings^38–40^; here, it denotes similarity^38,41^, that is, being another landowner). Peer influence is modelled as a social peer group network with landowners as network nodes, and environmental conversations as links between the nodes. The second influence type is *cross-scale influence,* which represents the persuasiveness of cross-scale actors (i.e. social actors who do not themselves make decisions to convert land) on landowners^14,20,42^. We include government representatives, councils, and indigenous groups as cross-scale actors. Connectivity and level of influence for peer influence network and cross-scale influence are self-reported by the respondents of the survey. Both peer influence and cross-scale influence links are weighted by the level of influence on landowners, as self-estimated by the respondents of the survey.

While peer influence and cross-scale influence are effected through conversations, spatial knowledge diffusion is mediated through behavior. *Spatial knowledge diffusion*^43,44^ occurs among contiguous neighbouring properties, and influences landowners’ decision-making through expressed enviromental behaviors and their visible outcomes^45,46^. This comprises a feedback from local changes in the biophysical environment to landowner behavior. In practice, the landowners in our model can observe changes in land use on adjacent farms; they then include this knowledge of their neighbors’ behaviors in their decision-making during subsequent years. Finally, habitat protection decisions are affected by a landowner’s personality traits, i.e. *actor attributes*. Each land-owner has a set of actor-level characteristics that influence his or her decisions about participation in environmental action (e.g., personal beliefs and farm characteristics^47^); these are called *actor attributes* when associated with social networks. Much empirical research has sought to identify the predictors of landowners’ adoption of conservation practices^47,48^. A suite of universal predictors that would enable targeting specific farmer profiles in conservation has not been identified; instead, such predictors are likely to be context-dependent^47,48^.

**Figure 2:**
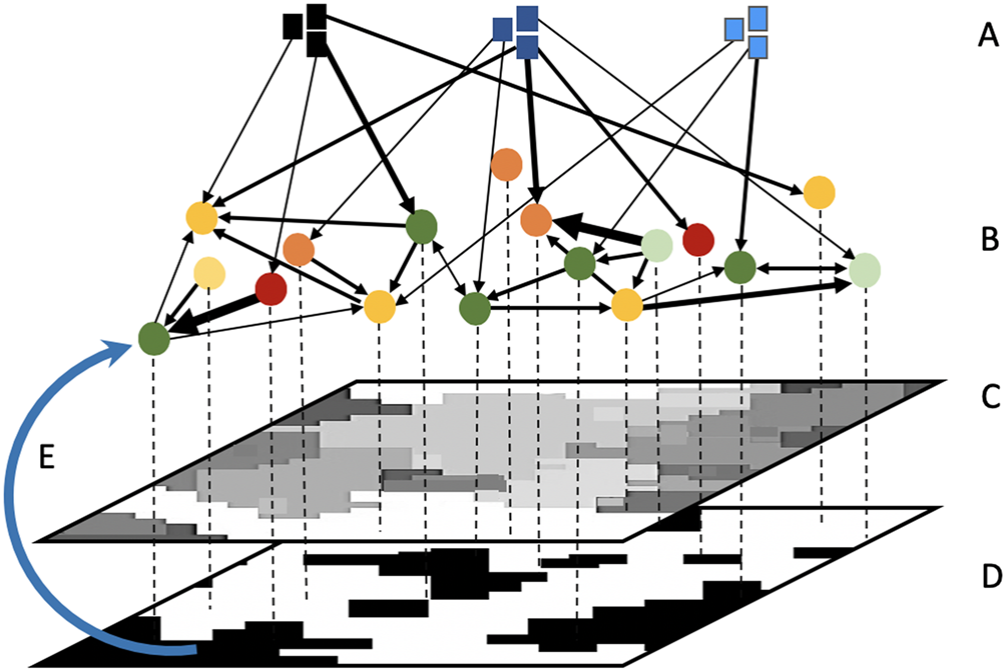
General model concept. The model consists of (**A**) three cross-scale actor groups and their influence links to landowners; (**B**) 200 heterogeneous landowners, each with his or her actor attributes, and influence links between landowners (peer influence); (**C**) a simulated agricultural landscape with areas available for conservation on each farm, upon which the landowner makes conservation decisions (dashed line); (**D**) a binary ecological landscape emerging from conservation action and consisting of either protected or unprotected land, coloured here accordingly; (**E**) spatial diffusion knowledge to each landowner from his or her neighbouring farms (here illustrated with one arrow only). (**A**,**B**) Network link weights represent the level of influence that landowners have self-reported their social connections to have. The figure was created using Microsoft PowerPoint version 16.43.

Using the survey data, we implemented a dynamic, social-ecological agent-based model to evaluate the impact of multiple, interacting social influences on the outcomes of conservation action on agricultural land (Fig. 2). We assessed the influence of the three social influence types of on landowners’ decision-making by varying the relative strength of each from them having no influence to being the sole influence on conservation decisions (Table 1), and modeled the spread of environmental behaviors under these different conditions. Quantitative knowledge is generally not available on the predictors of conservation decisions or their relative importance^47^. Thus, we systematically explored the plausible parameter space to identify which parameters are influential in the study context (a sensitivity analysis^49^). We then evaluated the consequences of landowners’ behavior on landscape structure by measuring resulting landscape-level protected area, habitat fragmentation and area of covenanted land, i.e. permanently protected habitat. The total sum of the influence parameter values (i.e. relative weights of each influence on decision-making) always sums to one. This approach prevents the model from creating unrealistic parameter combinations, such as two social influence types simultaneously having 0.8 influence on an individual. In so doing, our approach acknowledges that landowners are always affected by multiple influences. The individual effects of each influence on decisions can only be considered alongside other influences and landowners’ susceptibility to each influence. The only influence type that cannot have a value of zero is actor attributes; the landowners’ decisions are always influenced by their own characteristics.

**Table 1:** Model experiments.

Experiment	Parameter values
`Multiple influences experiment: Includes all social influences and actor attributes in decision-making Network model: actor similarity	Actor attributes: 0.1, 0.5, 1
	Peer influence: 0, 0.5, 1
	Cross-scale groups
	Indigenous: 0, 0.5, 1
	Council representatives: 0, 0.5, 1
	Government representatives: 0, 0.5, 1
	Spatial knowledge diffusion: 0, 0.5, 1
	Change-makers: 0.3, 0.7, 1
	Time steps: 0, 2, 6
Multiple influences (ER) experiment: Includes all social influences and actor attributes in decision-making Network model: Erdős Rényi	Actor attributes: 0.1, 0.5, 1
	Peer influence: 0, 0.5, 1
	Cross-scale groups
	Indigenous: 0, 0.5, 1
	Council representatives 0, 0.5, 1
	Government representatives: 0, 0.5, 1
	Spatial knowledge diffusion: 0, 0.5, 1
	Change-makers: 0.3, 0.7, 1
	Time steps: 0, 2, 6
Ingroup influence experiment: Includes peer influence and actor attributes in decision-making Network model: Actor similarity	Actor attributes: 0.1, 0.5, 1
	Peer influence: 0, 0.5, 1
	Change-makers: 0.3, 0.7, 1
	Time steps: 0, 2, 6
Ingroup influence (ER) experiment: Includes peer influence and actor attributes in decision-making Network model: Erdős Rényi	Actor attributes: 0.1, 0.5, 1
	Peer influence: 0, 0.5, 1
	Change-makers: 0.3, 0.7, 1
	Time steps: 0, 2, 6

In each experiment, the effect of social influences was tested by systematically changing their influence in decision-making. Landowners’ decision options include voluntarily keeping or converting part of their farm to protected habitat, either permanently or for the time being, or keeping or converting the land to productive use. Parameters are varied across plausible parameter ranges to detect which parameters are influential on conservation outcomes. The sum of parameter values for social influences and actor attributes is always scaled to one. “Change-makers” is the percentage of landowners making a decision during each time step. “Time steps” is the minimum time interval between land use changes.

The interplay of social influences in our study is not determined simply by the relative weights of each influence type on decision-making, but also by landowners’ self-reported individual differences in who they are influenced by and who they regularly interact with, as well as their actor attributes. Our study draws on extensive survey data, which produces actor diversity in the model in terms of the owners’ susceptibility to different social influences. Thus, allocating a high weight to a given social influence type does not necessarily mean that landowners will be influenced by it, or be influenced to an extent that affects the collectively produced environmental outcomes. It is the outcomes of the interactions in the model that are of interest.

To evaluate the influence of actor similarity-based landowners’ social networks, we used synthetic (i.e. generated by our model) social networks to perform actor-centric analysis^50^. In this analysis, the network plays an important role in the interaction between social actors, but different model outcomes are obtained by varying input parameters that are not related to the network itself^50^. A common approach to social network studies is investigating the extent to which peer influence and actor attributes explain environmental outcomes^e.g.^^9,51^. Here, in the context of simulation modelling, we call our experiments taking this approach ‘Ingroup Influence’ experiments as they include only peer influence and actor attributes in decision-making. We use the term ‘Multiple Influences’ experiments for simulations that include all three types of social influences and actor attributes in decision-making.

We conducted four in silico experiments (Table 1) to account for the influence of the network structure. The experiments were conducted with two synthetic, differently randomized peer influence networks. The actor similarity network is informed by survey data describing landowners’ interactions with different actor groups and self-perceived influence of these interactions. The network generation, therefore, captures social actors’ social network connectivity as a realistic number of links to other landowners and the influence of these links as self-reported in the survey. The second network is the Erdős-Rényi (ER) random network model^52^ in which landowners connect to each other at random at some fixed probability. We included the ER model for the purpose of determining the impact of actor similarity-based network on the environmental outcomes^50^. The ER model is not intended to capture characteristics of survey-informed social connectivity, but rather to serve as a null model against which to measure the impact of actor similarity-based network structure.

## Results and discussion

**Figure 3:**
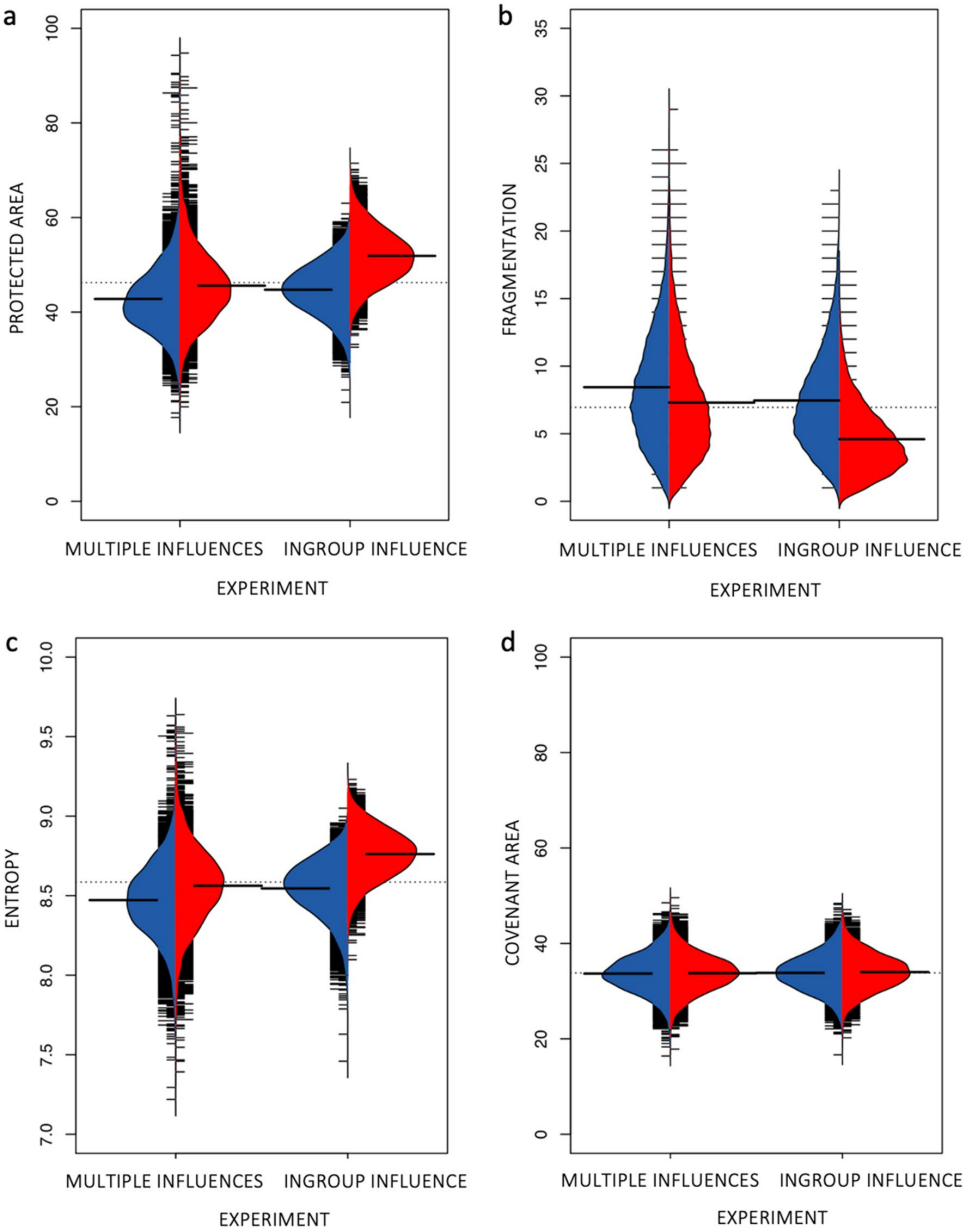
The main environmental outcomes for experiments including multiple social influences and peer influence only. The blue distributions present results for experiments using actor similarity-based network model, and the red distributions show results for experiments using ER model. Comparisons of experiment-specific outcomes are shown as bean plots. Horizontal black lines represent averages for experiment-specific distribution and dashed lines represent overall averages. (**a** and **d**) Show the total percentage of protected and covenanted area, respectively, of the land available for conservation in the modelled landscape. Fragmentation (**b**) represents the number of habitat fragments in the landscape and entropy the randomness of these fragments. The length of the bean per point found is 0.1. The high ends of the beans are cut to a maximum value of 0.2 for visibility of the distribution. The figure was produced using the Beanplot R package version 1.2^77,78^.

When decision-making is embedded in a dynamic SES, multiple types of social influence interact and can create unanticipated social-ecological dynamics. We detected a greater range of outcomes in landscape structure in experiments where landowner decision-making was affected by all three social influences compared with only peer influence. This result is evidenced by the vertical spread in Fig. 3a–c. A greater range of outcomes, including extremes in environmental outcomes, which in this context are success or failure in achieved conservation landscapes, emerged only when landowners were influenced by multiple social influences. For example, the Multiple Influences experiments produced landscapes where 18% to 95% of land was protected, whereas the Ingroup Influence experiments produced landscapes with protected area of 21–70% of available land (Fig. 3a). Similarly, on average we detected greater variability in habitat fragmentation in the Multiple Influences experiments than in the Ingroup Influence experiments. Thus, the Multiple Influences experiments more often produced unexpected outcomes than the Ingroup Influence experiments. Furthermore, the Multiple Influences experiments resulted in, on average, more desirable environmental outcomes (i.e. more protected land) than Ingroup Influence experiments (when using ER model), but this effect of multiple social influences was reduced when landowners formed social connections with other similar landowners (the actor similarity-based network model) (Fig. 3a–c).

**Figure 4:**
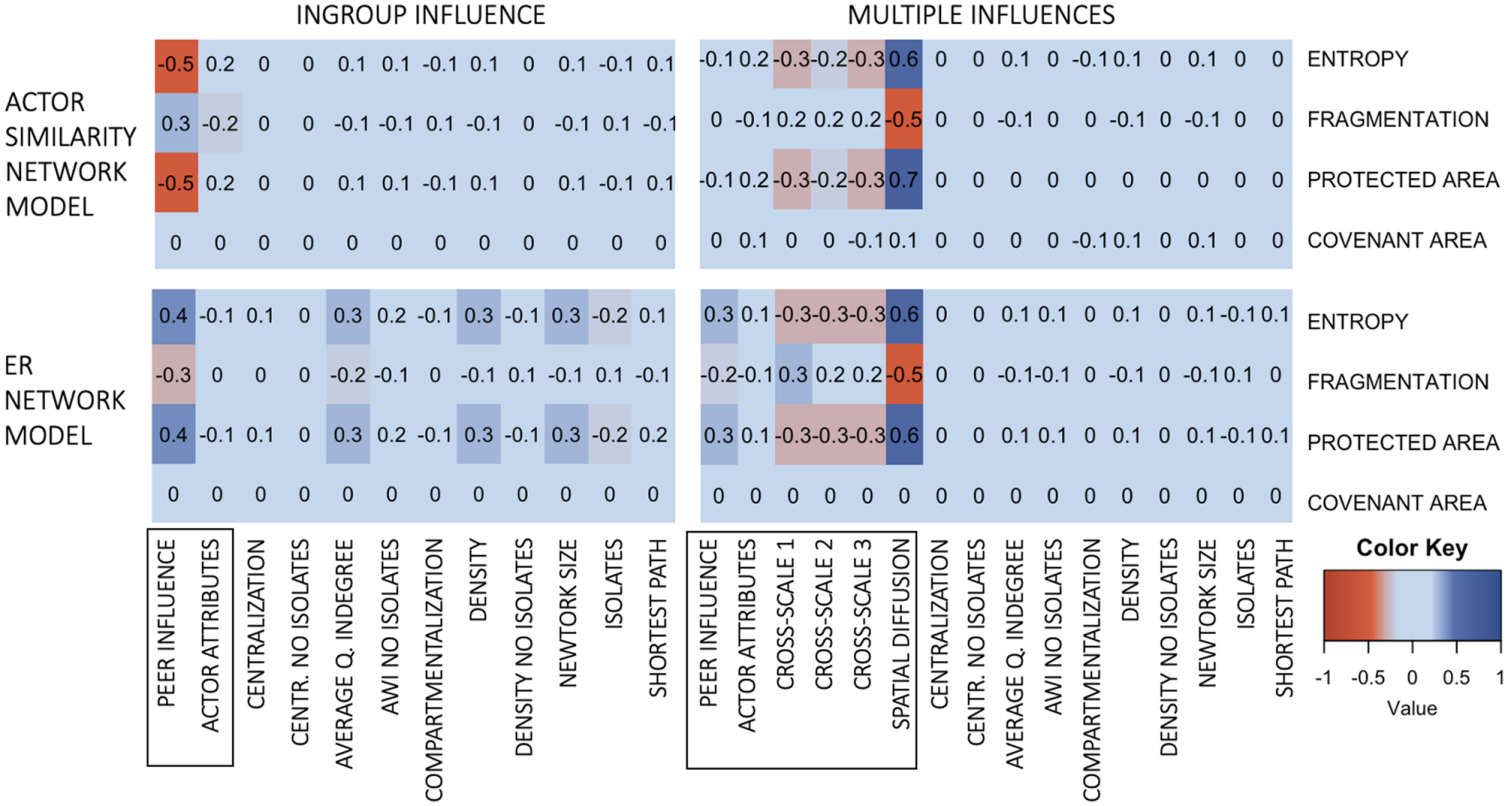
Experiment and model-specific correlation between environmental outcomes and factors that could influence environmental outcomes. Social influences and actor attribute included in decision-making are marked with a black rectangle, and the remaining variables on the y-axis are social network indices. The figure was produced using the ggplot2 R package version 3.0.3^77,79^.

**Figure 5:**
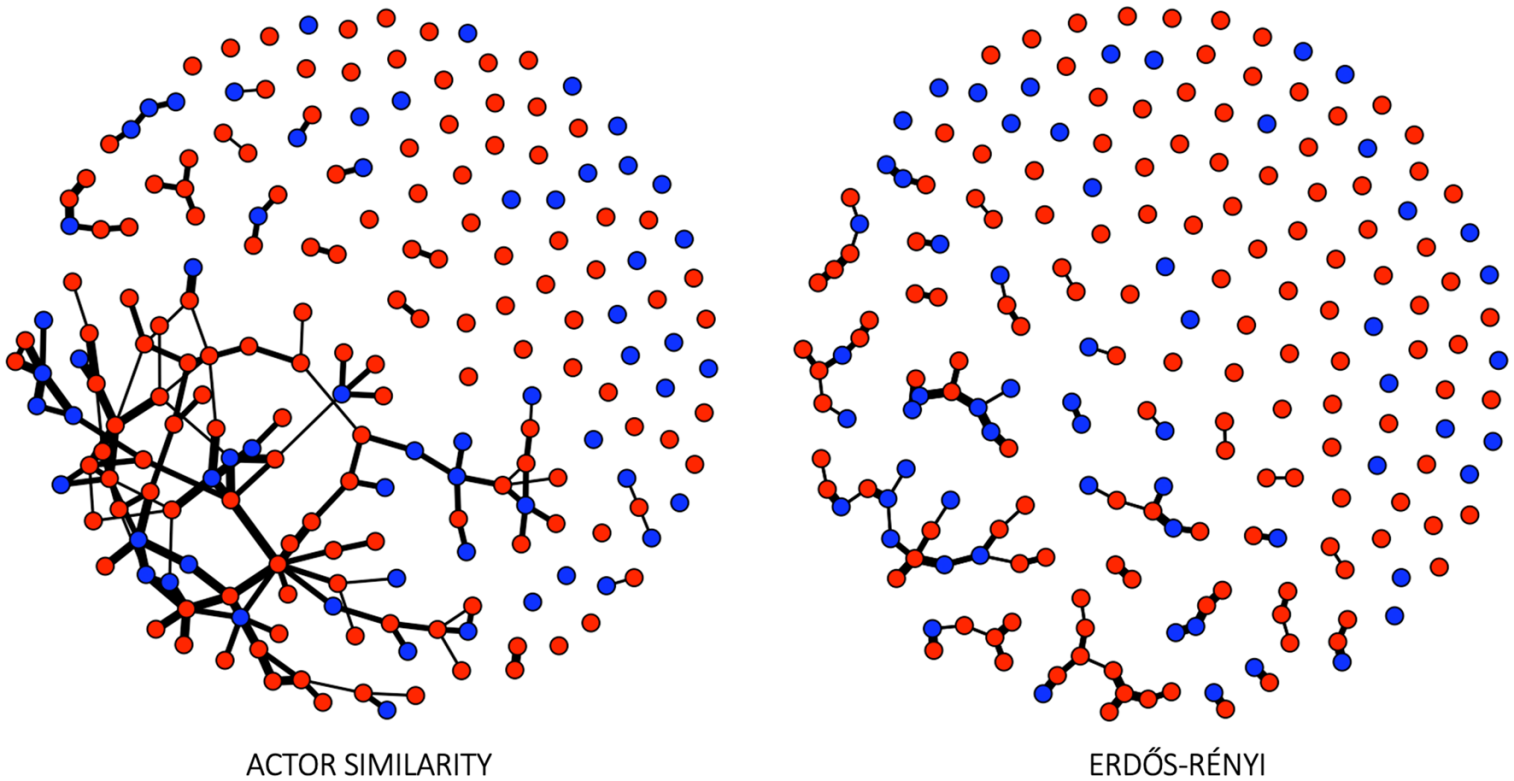
Peer influence networks for the two network models, each captured from one of the simulations with multiple social influences. Blue nodes represent landowners who have protected natural habitat on their land, red nodes are landowners without protected land. Note the mix of blue and red landowners in structures where network influence alone would have produced clusters of unicolor nodes. The isolates (unconnected nodes) represent landowners who did not report influential environmental conversations with other landowners. The network was visualized using the Fruchterman-Reingold layout in iGraph R package^77,80^.

When investigating which social or social-ecological processes in the model explain the experiment-specific differences in environmental outcomes, we found that (i) interacting effects of social influences create mechanisms that lead to accelerating change, and (ii) stronger social influence types can, in synergy or on their own, cancel the effect of a less dominant social influence. Our use of the term “strength” does not indicate only the relative weight of a social influence in decision-making, as per our analysis design; rather, a strong social influence is one that can affect and influence many landowners, even in the presence of other social influence types. These conclusions are based on the experiment-specific correlation (using Pearson’s *r*) between social influence and environmental outcomes (Fig. 4). First, our actor similarity-based and ER networks produced different results, demonstrating that different social influence types interacted with landowners’ peer group network structure (Figs. 3a–c, 4). When multiple social influences were included in landowner decision-making, spatial knowledge diffusion was the strongest predictor of environmental change. We interpret effect sizes (*r*) ≥ | 0.5 | as a strong association. This result is due in large part to the presence of a high number of landowners without network links to other landowners (i.e. isolates, Table 2) in our networks; spatial knowledge diffusion could directly affect all landowners, whereas peer group and cross-scale actor-influence directly affected only those landowners who had links with these groups. ER networks, which were generally more fragmented and contained more isolates than the survey-based networks, produced more desirable environmental outcomes (Table 2, Fig. 3a–c). This trend arises because landowners in actor similarity-based networks had more connections to others on average, and so had more potential to be influenced by their peer group than in ER networks. The typically cohesive structure of actor similarity-based networks allowed both undesirable and desirable behaviors to spread more effectively than in the more compartmentalized and fragmented ER networks (Table 2, Fig. 5). Thus, in the presence of strong spatial knowledge diffusion, social connections among like-minded landowners enabled peer group influence to mediate the spatial knowledge diffusion effect, producing ‘compromise’ environmental outcomes. In our study, both desirable and undesirable behaviors spread at the same time, and the typically cohesive structure of actor attribute–based networks allows both behaviors to spread more widely than in the more compartmentalized and fragmented random networks. Further, similarity among landowners was calculated using actor attributes. Thus, landowners who have a high (or low) probability of protecting land due to their attributes were connected to each other. Thus, altering behavior in such echo chambers would, in our study, require more behavioral diffusion than would altering behaviors of landowners who have connections to a more mixed group of landowners (random networks). Our model does not adjust the homophily-mimicking connectivity during the simulation. The landowners’ conservation behavior could become more diverse, but the network is not rewiring accordingly; like-mindedness is based on a number of actor attributes and not only conservation behavior.

**Table 2:** Social network indices.

Social network index	Actor similarity network	ER network
Network size (number of links)	91.000	74.000
	142.726	139.117
	217.000	212.000
Bridging actors	26	8.000
	46.272	30.565
	70.000	60.000
Isolates	37.000	61.000
	69.262	99.657
	101.000	136.000
Compartmentalization	0.210	0.791
	0.677	0.934
	0.911	0.974
Average weighted indegree without isolates	0.523	0.642
	0.723	0.918
	0.979	1.225
Density	0.002	0.002
	0.004	0.003
	0.005	0.005
Density without isolates	0.006	0.010
	0.008	0.014
	0.012	0.021

Calculated from networks for both network models, a total of 13,122 simulations (6561 each). Density was used in network randomization in Erdős Rényi (ER) network model experiments. For each index, the table shows the minimum value, the mean value and the maximum value for all simulations, in respective order. The full table and descriptions for each index can be found in Supplementary Materials tables S3 and S4.

To describe the structure of landowners’ peer influence networks, we measured a number of network indices, which have been found to be influential for environmental action in empirical and theoretical research (Table 2). We found moderate effect sizes (*r* > |0.3|) for structural network properties only in the Ingroup Influence experiment conducted with ER networks, which might suggest that network structure did not influence environmental outcomes in other experiments. However, our results do not support this interpretation. The differences in environmental outcomes between the actor similarity-based network and the ER network demonstrate that network structure strongly influences the environmental outcomes in our model. The small effect of network structure shown in Fig. 4 is more likely due to the presence of stronger social influences (Table 2). Using the actor similarity-based network, which was generated to capture social connectivity and influence among landowners as self-reported in the survey and was based on like-mindedness, led, on average, to less-protected landscapes with more habitat fragmentation. Hence, network structure is another important factor in mediating environmental behavior in the presence of multiple types of social influences. However, measuring network indices could not disentangle *which* structural characteristics benefit environmental outcomes; future studies should test generating different network models and explore the association between modifications of network structures and environmental outcomes.

Since individual actor attributes did not influence environmental outcomes (effect sizes r > |0.3|) and a greater range of outcomes were detected in experiments with multiple social influences, the extreme outcomes emerged from the combined effects of spatial knowledge diffusion and peer influence. The results suggest that the combination of these two social influence types produced a social-ecological dynamic that generated accelerating gain or loss of natural habitats. While spatial knowledge diffusion produced spatial clusters of protected or unprotected areas, behavior in peer influence networks spread independently of landowners’ spatial locations. Behavioral change through peer group influence networks could therefore ‘jump’ in space and produce protected areas in otherwise unprotected regions, or vice versa, which then seed new clusters induced by spatial knowledge diffusion. The process is similar to the core-satellite spatial pattern seen in ecological invasion due to long-distance dispersal^53^. The detected social-ecological spatial dynamic is facilitated by more fragmentation and spatial habitat clustering (i.e. lower entropy) in Multiple Influences experiments, especially with actor similarity-based networks, which have fewer isolates. This combination allows more seeds to emerge that interact with social network diffusion. In real systems, inertia effects, such as delays in creating or detecting local environmental change^34^, may slow change driven by such social-ecological mechanisms.

Furthermore, the results indicate that the strong combined effect of spatial knowledge diffusion and peer influence cancelled out that of cross-scale actors. Although in our model we assume a positive influence from cross-scale actors on landowner decisions to protect land, desired environmental outcomes occurred less often as the cross-scale actor influence on landowner decision-making increased (negative correlation in Fig. 4). That the weighting of other stronger drivers must decrease as the influence of cross-scale groups, which each had their own weighting, increases (because all influences were scaled to collectively sum to one), may explain the negative effect of these cross-scale groups. However, the relative weight of each influence is mediated by the social-ecological context of the decision-making, such as landowners’ self-reported connectivity and susceptibility to cross-scale actors, or spatial patterns of the landscape. It is most likely that the weak and negative influence of cross-scale actors was due to the low number of landowners connected to cross-scale actors. In our sample of 600 landowners, only 11 (1.8%) reported influential environmental conversations with indigenous groups, 143 (28.3%) with local councils and 18 (3.0%) with central government representatives.

Finally, we included actor attributes both as a separate driver in decision-making and through actor attribute-based similarity in the survey-based network construction. Hence, connectivity in the actor similarity-based networks propagates the influence of actor attributes.

None of the social influence types correlated with the area of covenanted land in any of the experiments (Fig. 4). Since covenanted land cannot legally be unprotected and returned to agricultural use, increases in covenanted areas in our model were mainly influenced by the extent of covenanted areas at the beginning of the model simulations (Supplementary Materials, Figure S1a–d). This outcome implies that in the model the landowners rarely made the decision to covenant land. The extent of conserved and covenanted areas at the beginning of the simulations was determined by survey responses of the landowners randomly selected for each simulation, contributing to the initial landscape composition being aligned with the characteristics of landowners in the experiments.

Unanticipated environmental outcomes in social network studies can result from treating social networks as “closed systems”, i.e. failing to consider social influences from outside the network under study. In our study, inclusion of multiple social influences in landowner decision-making increased the variety of collectively achieved environmental outcomes and led more often to extreme environmental outcomes than a setting where only actor attributes and peer influence affected land-owners’ decision-making. Importantly, our study suggests that the effects of multiple social influences, when included in analysis of an SES, should not be assumed to be additive. These effects are mediated by social network structure, actor diversity and the presence of other types of social influences. In this context, multiple social influences may interact antagonistically or synergistically and, in so doing, create unanticipated social-ecological mechanisms for environmental change. Consequently, the presence of multiple social influences can create more unpredictability in emerging environmental outcomes than when only one social influence (e.g. peer influence) is included in individual decision-making. Detecting these effects requires situating environmental decision-making in a dynamic social-ecological context in which human behavior and its context are ever evolving and influencing each other^cf.^^15^.

Numerous studies of social networks representing a single type of connectivity have linked network structure to environmental behavior or outcomes^9,12,54^, but few studies have measured the effect of micro-scale social interactions on environmental outcomes^55^. Although based on a national survey of New Zealand landowners and land managers, our model provides generalizable insights on potential social influence leverage points for conservation. The strong influence of spatial knowledge diffusion suggests that visible pro-environmental behavior^46^ provides a feedback between the ecological and social subsystems, which could change the behavior of people who lack social connections or whose social connections may not promote pro-environmental behavior. In so doing, spatial knowledge diffusion can produce spatial clusters of conservation activity that would benefit biodiversity and other environmental conditions. Purposefully establishing such ‘seeds’ of conservation could trigger willingness of others to adopt pro-environmental behavior(s), especially if seed landowners commit to long-term conservation via mechanisms such as legally binding covenants.

A social network with stronger peer influence links and fewer isolates could, in another setting, outweigh the influence of spatial knowledge diffusion. Intriguingly, a social-ecological feedback loop including environmental change, emergence of clustered protected areas or strong influence links between spatially decoupled landowners, could potentially provide early warning signs for accelerating landscape-level change. Alternatively, the pattern could provide an opportunity for network intervention^56^. For example, landowners encouraging their neighbors to undertake private land conservation integrates both ecological feedbacks and peer group network influence. This type of intervention has recently been tested with successful outcomes for landscape-level conservation^36^. In our study, the inclusion of multiple social influences and simultaneous spread of undesired and desired behaviors likely hindered clusters of behavior from emerging in ingroup networks (Fig. 5). However, we did not assess the presence of behavior-based clusters in our analysis as the aim was to measure environmental outcomes of landowners’ behavior.

Our results are based on allowing undesired and undesired behaviors to spread simultaneously. This context, together with the interplay of multiple social influences, will produce uncertainty in social network interventions. Identifying change agents (e.g. opinion leaders^56^) based on their network position is only the first step in using network interventions to accelerate behavior change. The ability of change agents to trigger behavioral change also depends on their positions in a wider, evolving social-ecological context^57–59^. Furthermore, while involvement of cross-scale actors in environmental decision-making commonly increases the diversity of information in the network, relying on them to encourage pro-environmental behavior may be insufficient if they have few strong connections to landowners.

Finally, the important role played by isolates in our study highlights the need for careful setting of network boundaries^60^, i.e. who to include as network actors when preparing social network research or interventions. Snowball data collection methods^60^, for example, may lead to the inappropriate exclusion of isolates, as the sampling technique is based on recruiting acquaintances of network members. Network influence research resulting in unexpected environmental outcomes may benefit from testing the boundaries of social networks under study. Considering information flows from multiple sources is important, especially when social actors make decisions that require social reinforcement in the form of social norms or demonstration of benefits, in contrast to situations where behavioral change is easy and non-costly^19^.

Our model is necessarily a simplified representation of decision-making in SES and SES dynamics. We assumed that all landowners can allocate a fraction of their land to conservation, and we do not consider temporal changes in social or economic conditions, or habitat quality. Our representation of spatial knowledge diffusion is based on the idea that social norms and/or demonstration of conservation action generate a reinforcing feedback. However, a balancing feedback may also result from a decrease in protected areas triggering pro-environmental behavior as landowners observe an increased need for conservation^61,62^. While using synthetic social networks is common practice in agent-based modelling when research questions are difficult to test with other methods or empirical data are limited^50,63^, this approach may influence our results as the structure of our actor similarity-based network varied only modestly. Furthermore, our actor similarity-based network model generation might not capture all topological features of real-world networks, and we therefore can only conclude that this mechanism matters in the context of our study, without identifying the network structural or other pathways through which this effect is realised. To address this caveat, future research should test network-centric or structurally explicit analyses^50^. Such analyses could show that specific network structures, such as clustering, significantly influence behavioral diffusion in a social network even under multiple social influence types. Assessing the influence of network structures and the location of specific social actors in the network could enable the detection of causality between different social influences and social network structures. For example, if most landowners with connections to cross-scale actors tended to be clustered together with few links to other landowners, then cross-scale influence would be limited at landscape extents. Although these issues remain unexplored in our study, the identification of these caveats through our research highlights the importance of developing complex simulation models to better understand how social networks function when embedded in social-ecological dynamics.

To facilitate comparison with previous social network research, we treated the networks as static, although complex adaptation in SES will most likely include rewiring of social influence links and learning that changes the strength of influence among social actors. Studies that allow the social network structure to adapt to a changing social and ecological context during the simulation are needed. We also hope to see multi-level and multiplex network approaches adopted in studies considering multiple social influences in SES^e.g.^^64,65^. Finally, by scaling the total influence of the behavioral drivers to always sum to one, we assumed that there is a maximum extent to which individual decision-making can be influenced. Hence, an increase in the importance of one driver results in a commensurate decrease for the others, which may explain the negative correlation between some drivers and environmental outcomes in the presence of strong drivers.

Studying social networks as part of larger social-ecological frameworks and drawing on interdisciplinary theory will improve our understanding of complex human and environmental dynamics. A critical step in this process is considering social influence networks as open systems that interact with other networks and the decision-making environment^15,66^. It is known, for example, that failure of nodes in one network leads to the failure of nodes in other networks through dependency links (e.g. problems in financial systems cascade to work places and, through unemployment, to families)^66,67^. Our study provides a step in this development path by considering the interplay of different types of social influences, embedding behavioral decisions into a dynamic SES context and evaluating the environmental outcomes of social influences with biodiversity-relevant landscape indicators.

## Conclusions

Our research emphasizes how the presence of multiple social influence types can produce unexpected environmental outcomes in environmental decision-making. Likewise, to understand links between social influence networks and environmental outcomes it is important to consider social influence as embedded in complex adaptive social-ecological systems, in which human behavior consistently adapts to changing social and ecological contexts. Social networks are not closed systems, but rather have potentially important feedbacks between the environment and different components of the social system. Considering social networks as adaptive elements of complex and dynamic social-ecological systems will improve our capacity to fully understand how social influence contributes to generating desired environmental outcomes.

## Materials and methods

### General model concept

We developed an agent-based model to evaluate the impact of multiple, interacting social influences on landowners’ conservation action on agricultural land, and consequently on landscape-scale environmental outcomes (Fig. 2). A detailed Overview, Design concepts and Details (ODD) protocol (Grimm et al.^68^) of the model is available in the Supplementary Materials (SM). Data for the study were collected in the 2015 Survey of Rural Decision Makers^37^, which is a large, internet-based survey covering more than 3300 farmers across all primary industries and regions of New Zealand. Due to question randomisation and survey branching, the usable data set for this survey included 600 private landowners and land managers involved in primary production. Isolates may emerge in survey data collection^e.g.,^^69^ and we retained them in our study networks because of their potentially important role in environmental or resource collectives.

Model simulations began with 200 landowners, randomly selected for each simulation from the 600 landowners with complete survey data. These landowners were assigned at random to 200 farms on the model landscape. At the start of each simulation, protected natural habitat was present only on the farms of the landowners who reported having native forest or covenanted land. During each time step, a changing subset of landowners decided whether to protect natural habitat on their land; if they decided to protect the land, they also decided whether to covenant. (Covenanting land is a practice increasingly adopted by landowners in New Zealand. It is an agreement between a private landowner and the QE II National Trust to protect land, even if the property is sold to a new owner^70^). Landowners with self-reported barriers, such as fear of losing rights to own land, could not commit land to covenants. Landowners could decide against protecting land only if the habitat was not covenanted. The conservation landscape and conservation status of each landowner were updated according to landowners’ environmental behavior, so that during the subsequent time-step decisions took place in an updated social-ecological context. We simulated a period of 150 time-steps, which represents approximately 50 years. The model was run for a 50 time step burn-in period before data were collected.

The landscape component of the model was represented on a toroidally wrapped grid, i.e. a lattice. Each cell in the landscape could occupy one of three states: protected, unprotected, or covenanted. For habitat connectivity variables (number of habitat fragments, entropy), connected protected cells formed a non-fragmented habitat area; any non-protected cells between protected patches indicate the presence of habitat edges. Because our model landscape consists only of areas available for conservation, the percentages discussed in the study are not directly comparable to suggested critical thresholds in habitat declines that lead to abrupt biodiversity losses, e.g.^71^. We chose to model the land available for conservation, which we set to be 10% of each land-owner’s land with an assumption that the farm would remain financially viable. Accordingly, the model landscape consisted only of land potentially available for conservation (i.e. only land where at least partial protection for conservation is a feasible option), and is subdivided into farms owned by the 200 landowners represented in the simulation. Hence, fragmentation was determined relative to the maximum possible area, given the availability of farmland for protection. The amount of land available for conservation (10%) was arbitrary but was fixed across experimental treatments. This simplification avoided the possibility of unlikely outcomes such as landowners protecting 100% of their land, while allowing us to avoid further complicating the model by including economic processes and parameters. We assumed that the extent to which landowners prioritise profit over conservation are captured by the actor attributes, which were measured in the survey. The size of each farm was based on the survey data, scaled to be consistent in every run.

To determine a set of actor attributes that could influence native habitat protection, we performed logistic regression analyses on variables from the survey that described landowners’ views and values for conservation and covenants, their farming industry, land-use and whether they live on the farm (Supplementary Materials Table S5a–d, a detailed examination of the diversity of survey respondents can be found in^13^). The set of 28 variables (Supplementary Materials Table S7) included in the regression was used to calculate pairwise Gower’s dissimilarity^72^ for the 600 landowners (to be used in network generation below), which was used to construct actor similarity-based networks.

In peer group social networks, nodes represent landowners and directed links represent influential environmental conversations between peers. Each land-user’s in-degree and link weight were reported in the survey as the number of landowners with whom they had environmental conversations and a categorical evaluation of the influence of these conversations (SM: Network Questions), respectively. We removed links for which the level of influence was reported as “not influential”. Because the survey captured the number and level of influence but not the identity of influence partners, we evaluated two methods for allocating these influence links to other landowners: Erdős–Rényi (ER) randomization^52^ or link allocation based on actor similarity to mimic homophily. Homophily is influence-based contagion driven by similar people adopting similar ideas and, over time, actor attributes can become correlated with the structure of social networks^10,73,74^. In actor similarity-based networks, like-minded landowners influence one another, such that the probability of each pair of landowners (with indegree > 0) being connected was inversely proportional to the dissimilarity in their attributes from the survey. As a null comparison against these networks, ER networks allocate links at random according to the Erdős–Rényi random graph models^52^. We used the mean link density of > 6500 model-generated actor similarity-based networks (0.0035) as the probability of assigning a link between any two landowners in the ER networks. In ER networks, categorical weights representing slight/moderate/high influence were assigned to each link at random, whereas in actor similarity-based networks, the link weights are those self-reported by landowners for each social group in the survey. We included three cross-scale groups, which were included in the survey: central government representatives, local council representatives and an indigenous group. Links to cross-scale groups and their influence were reported by survey respondents as with peer links. In both network structures, the number of nodes was fixed at 200.

### Simulations

The effect of each type of social influence or actor attribute on decision making was scaled to sum to one (Table 1). To determine sensitivity of the results to model structure, and since it is unlikely that landowners would frequently change land-use, we varied the percentage of landowners who make a decision during each time-step (30, 70 or 100%) and the minimum time interval between land use changes (0, 2 or 6 time-steps) for each parameter combination. One simulation was run for each parameter value combination for the experiments, including all social influence types or actor attributes, resulting in 6561 simulations per experiment. Ingroup Influence experiments (which had fewer unique combinations due to fewer drivers) were run with repeated simulations (*n* = 75) to total 6561 and have a consistent number of simulations for each experiment.

### Land-use decision making

Each landowner’s decision to protect, or unprotect, habitat on their land was calculated using the weighted sum of the factors included in the decision making. Each social influence or actor attribute had a value between 0 and 1, with higher values indicating a higher likelihood of protecting land. Peer influence indicates the number and influence (weight) of links that a landowner had to other landowners across all the actor’s weighted links; it is based on weighted indegree and was calculated for actor *i* as:

1 $$C_d(i)=\frac{\sum _{j=1}^{n_c}x_{ij}w_{ij}}{\sum _{j=1}^nx_{ij}w_{ij}}$$

where *n* is the number of nodes in the network, *n*_*c*_ is number of nodes currently conserving habitat on their land, *x* is the value of the link (1 if the nodes are connected) to actor *j* and *w* is the link weight.

The influence from each cross-scale actor group was calculated relative to the maximum cross-scale influence (*C*_*max*_) in the land-owner network:

2 $${C}_{cs}\left(i\right)=\frac{k{w}_{c}}{{C}_{max}}$$

where *k* is the landowner’s in-degree to that cross-scale group and *w*_*c*_ is the influence of those links (both derived from survey data).

The spatial information influence for respondent *i* was calculated as:

3 $$E\left(i\right)=\frac{{N}_{c}}{N}$$

where *N*_*c*_ is the count of adjacent farms with protected habitat and *N* is the total number of adjacent farms.

Respondent attribute influence was calculated from a logistic regression with the probability of protection native forest as the outcome variable and survey responses as predictors (*X*):

4 $$P\left(Y\right)=\frac{1}{1+{e}^{\left({\beta }_{0}+{\beta }_{1i}{X}_{1i}+{\beta }_{2}{X}_{2i}+{\cdots \beta }_{n}{X}_{n}\right)}}$$

where *β*_*n*_ is the regression coefficient for variable *X*_*n*_.

The probability of land being covenanted was calculated in a similar way to land protection, with the exception that if the respondent had reported reasons for not covenanting land (e.g., no suitable land available on farm or concerns over covenant regulations or losing the right to change covenanted land), they would always decide against it.

Finally, in our representation of decision making, the influence of each social influence or actor attribute is weighted by the landowner’s individual parameter values. The probability of a landowner protecting land is the weighted sum of *n* behavioral drivers:

5 $$P(protect)={\sum }_{j=1}^{n}{y}_{j}{f}_{ij}$$

where *y*_*j*_ denotes the weight (parameter value in our model) of importance of each social influence type or respondent attribute in decision -making, and *f*_*j*_ denotes the value of the influence.

### Data and software availability

We used NetLogo 6.0.3.^75^ for model programming and simulations, including the R extension^76^, and R Studio version 1.1.463 environment for supporting coding and analysis^77^. Pseudocode for the model and needed data input files for the model are available in Supplementary Materials, including a sample data for actor attributes. The full dataset can be requested from the authors with consideration to survey respondents’ anonymity. Simulated, simplified landscapes and subsamples of landowners make the survey respondents unidentifiable in the model.

## Supplementary Information

Supplementary Information 1.

Supplementary Information 2.

Supplementary Information 3.

Supplementary Information 4.
