# Supplementary material for: Multiple social network influences can generate unexpected environmental outcomes
Source: Sci Rep. 2021 May 7;11:9768. doi: 10.1038/s41598-021-89143-1 (PMC8105375; doi:10.1038/s41598-021-89143-1)
Supplement: Supplementary file 1 — Supplementary Information 1. [file 41598_2021_89143_MOESM1_ESM.docx]

SUPPLEMENTARY INFORMATION FOR:

MULTIPLE SOCIAL NETWORK INFLUENCES CAN GENERATE UNEXPECTED ENVIRONMENTAL OUTCOMES

Yletyinen, J.*, Perry, G.L.W., Stahlmann-Brown, P., Pech, R., Tylianakis, J.M.

* Corresponding author: yletyinenj@landcareresearch.co.nz

**This file includes:**

Supplementary text: Network questions

Figure S1a-d. Baseline protected and covenanted area

Table S1. Survey - based actor attributes and landscape variables

Table S2. Environmental outcome variables

Table S3 Structural social network indicators: definitions

Table S4 Structural social network indicators for actor similarity-based and ER networks

Tables S5a-d. Logistic regression results for actor attributes

Supplementary Materials Appendix A**:** Overview, design concepts and details

(ODD) protocol for Landowner model

Table S6 Model input data: actor attributes (online)

Table S7. Model input data: actor similarity matrix (online)

Table S8. Input data for calculating Gower’s similarity (online)

Supplementary references

Supplementary Information Text:

**Network questions in the Rural Decision-Makers 2015 Survey**

This section describes how the *Rural Decision-Makers 2015* survey data ^1^ were converted to a social network structure. In the social network including only landowners, each node is an individual landowner, and each link represents influence mediated by conversations about environmental issues. First, landowners were asked "*Did you regularly meet with individual people from the following groups to discuss environmental performance of your farm business over the past 12 months*?" If a landowner chose "*Farmers in your industry" or "Farmers in different industries",* they received two additional questions on the number and influence of connections they have to other landowners. The in-degree (number of incoming links) is based on the landowners’ reply to the question: *"With approximately how many individuals from each of the following groups did the trust board regularly meet to discuss environmental performance of the farm business during the past 12 months?".* Then, the landowners evaluated the influence (*"How influential is advice about environmental performance from these individuals?")* on four-categorical scale: not at all influential, slightly influential, moderately influential, extremely influential. The answers were quantified and standardized to numeric link weights (0, 0.33, 0.66, 1). If a landowner replied "*Not at all influential",* the link weight becomes zero and the landowner’s in-links to other landowners are removed from the network.

The second level of the network, i.e., landowners’ links to stakeholders, include survey-based data on the indegree (i.e. connectivity between a landowner and representatives of New Zealand Māori iwi). The landowners were also able to select stakeholder groups in the question "*Did you regularly meet with individual people from the following groups to discuss environmental performance of your farm business over the past 12 months*?") and influence (*"How influential is advice about environmental performance from these individuals?").* We did not include stakeholders as nodes in the model networks since it would have required inventing the total number of cross-scale actors available (the model does not represent any specific area) and we do not know whether the landowners have talked to the same or different individuals in stakeholder groups. Thus, we avoided making assumptions about the topology of intermediate social network linking the landowner and stakeholder levels. Instead, cross-scale actor data is stored as landowner variables.


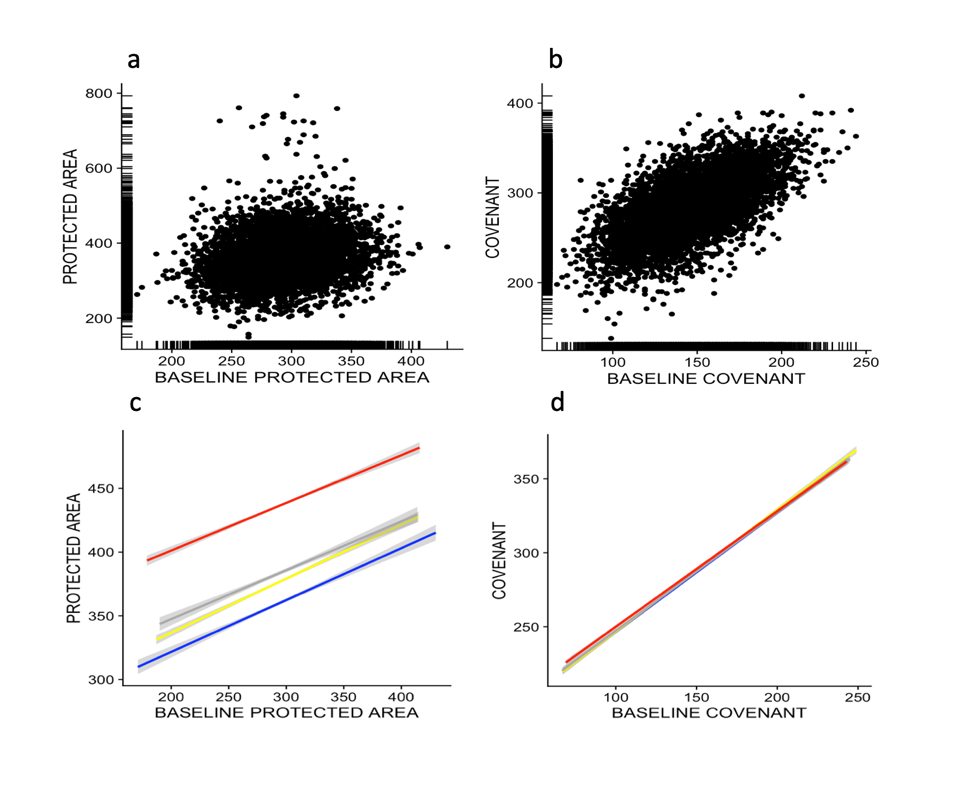


**Figures S1a-d. Relationship between baseline habitat and environmental outcomes.** Due to diffusion processes (spatial knowledge diffusion, social network influence), the extent of protected area in the beginning of model simulation affects the extent of protected area and covenanted area in the end of model simulation. (a) shows the relationship between the extent of protected area in the beginning (baseline) and at the end of model simulations, for the experiment including an actor similarity – based network and multiple social influences. The x- and y-axis units represent number of protected grid cells on the landscape model (total 841 cells). (b) shows the same relationship for covenants, suggesting that the relationship is more linear because covenanted land cannot be unprotected. Note that in figure a, the most successful protection cases occur with circa 250 – 350 baseline habitat area, not the highest. Figures c and d shows the same trends as regression lines to illustrate experiment-specific differences, with confidence interval 0.95. The blue and gray lines represent Multiple Influence experiments with actor similarity - based and ER network models, respectively. Yellow and red lines represent Ingroup Influence experiments with actor similarity - based and ER network experiments, respectively. Contrary to protected area, experiment-specific differences in the resulting extent of protected area were not detected for the increase in covenanted areas.

Table S1. Survey-based actor attributes and additional landscape variables included in the model. Prefix 's' in front of the ID number indicates Rural Decision-Makers survey - based variables. Variables calculated in the model can be found in Table S2 (prefix 'm'). In the Variable column, a letter indicates the reply options for each survey question: (B) binary (yes/no) reply, (C) categorical reply, (N) a value reported by the landowner. We assume any additional variables to be constant across the landowners throughout the modelled time period. These variables may include economic (e.g., market forces), political (e.g., policies that increase the productivity of the land and make setting aside land for conservation a greater loss) and institutional factors (e.g., attitudes on the public having right to recreational natural spaces).

| ID | VARIABLE | DESCRIPTION |
| --- | --- | --- |
| **Land use and natural environment on the farm** | | |
| s1 | Land use (C) | Land use on the farm. Selecting multiple options was allowed in the survey; in the model, each land use is a variable of its own. Land use options:  - grazing livestock  - operating a dairy platform  - operating a dairy run off  - farming sheep and or beef  - raising and/or finishing prime cattle, including bull beef  - raising deer  - raising pigs  - raising poultry birds  - raising other farmer livestock  - growing grain and seed crops  - growing crops for hay, silage or balage  - growing vegetables and/or cooking herbs indoors  - growing vegetables and/or cooking herbs outdoors  - growing flowers, bulbs, nursery crops, or hops  - growing kiwifruit  - growing wine grapes  - growing other fruits, nuts, or edible tree crops  - plantations of exotic trees inteded for harvest  - harvested exotic forest area awaiting restocking  - farm-based tourism  - wetlands  - **native forest and shrubs and/or tussock grasslands: farm land contains native forest and shrubs and/or ungrazed tussock grassland. This variable was used as an indicator of protected land on a farm, and as an outcome variable in regression analyses.** |
| s2 | Covenant (B) | All or part of the land is covenanted, i.e. permanently protected. |
| **Farming characteristics** | | |
| s3 | Primary industry (C) | Landowner's primary activity, i.e., how the landowner primarily identifies him/herself as a farmer. Selecting multiple options was allowed in the survey. Primary activity options:  - farming sheep  - farming beef  - farming sheep and beef  - dairying  - deer farming  - grazing livestock not owned by the farming business  - pig farming  - poultry farming  - other farmed livestock  - arable farming  - vegetable production  - growing flowers, bulbs, nursery crops, or hops  - kiwifruit production  - wine grape production  - growing fruits, nuts, or edible tree crops  - exotic forestry  - farm-based tourism  - native forest and shrubs and/or tussock grassland  - other |
| s4 | Ownership (C) | Ownership of the land: myself (landowner) / another individual / family partnership / family trust / family company / Māori trust or inc. / corporate owned / equity partnership / family company |
| s5 | Live on farm (C) | The landowner lives on the farm: year-round / part of the year / not at all. |
| s6 | Area (N) | The total area of the farm in hectares. This variable addresses the largest land parcel of the farm, even if the farm includes additional blocks. |
| **Personal values** | | |
| s7 | Private conservation (C) | Private landowners should protect habitat for native plants and animals on private land: strongly disagree / middle / strongly agree. |
| s8 | Public conservation (C) | The New Zealand Department of Conservation should protect habitat for native plants and animals on public land: strongly disagree / middle / strongly agree. |
| s9 | Covenant barriers (C) | The reasons why landowner has not joined a covenant: don't have suitable land / fear of losing property rights / too much regulations. In the model, each reason is a variable of its own. |
| **Social networks** | | |
| s10 | Indegree (C, N) | The number of connections a landowner has to other landowners and to each cross-scale actor group. This variable consists of two parts: i) whether the landowner has regularly met to discuss environmental performance of the farm with representatives from the following actor groups (C); and ii) with approximately how many representatives of each group they have met regularly (N). Indegree for any of the stakeholder subgroups can be zero if a landowner has no connections to that group. The model includes following network actors:  - regional councils, district councils  - central government  - iwi, i.e. largest social units of māori in New Zealand, often translated to “tribe” in English. In this study, we call iwi “indigenous groups”.  - farmers in the same industry as landowner  - farmers in different industries than landowner |
| s11 | Social influence (C) | Landowner's estimation of how much influence his/her social connections (see list on the stakeholder subgroups in variable s10) have on him/her: not at all influential / slightly influential / moderately influential / extremely influential. If the connection is "not at all influential", landowner's degree to that actor group becomes zero in the model. |

**Table S2. State variables included in the study**.

| **Variable** | **Description** |
| --- | --- |
| Baseline protected area | The extent (number of patches) of protected area in the beginning of simulation |
| Baseline covenant | The extent (number of patches) of covenanted area in the beginning of simulation |
| Protected area | The extent (number of patches) of the total protected area in the landscape, measured at the end of simulation |
| Covenant area | The extent (number of patches) of the total covenanted area in the landscape, measured in end of simulation |
| Fragment count | The number of natural habitats fragments (a fragment is defined as a protected area surrounded by land in other use) at the end of simulation |
| Entropy | A measure (Shannon entropy, ^2^) of spatial randomness / arrangement of the natural habitat fragments in landscape, measured at the end of simulation |
| Residence time | The total length of time (number of time steps) that land was protected, even if discontinuous. The average protection time was calculated from a list of protection times for each patch, and it excludes 50 first steps for model initiation. |
| Conservationists | The number of landowners who have protected native forest, calculated in the end of simulation |
| See Supplementary Materials Tables S3 and S4 for social network analysis indices. | |

Table S3. Structural social network indices. The indicators included in the

study were chosen based on influence on collective action or environmental management described in previous studies. Due to the high number of isolates in our networks, some indices were calculated with and without isolates.

| SOCIAL NETWORK INDICES | |
| --- | --- |
| INDEX | RELEVANCE |
| **Mean path length**: the average of shortest paths between all actor (node) pairs ^3^ | A short mean path indicates that everyone in a network is fairly 'close' to each other, and actors can thus reach each other through a small number of intermediate actors (cohesive network) ^3^. |
| **Density**: the ratio between the actual number of links in a network and the number of possible links ^3^ | Density affects collective action as increased possibilities for communication (influence), exposure to new ideas and knowledge ^4^. However, very high density may hinder efficiency of collective action, or lead to homogenization ^4^. In influence network, short mean path and density would thus lead to quick spread of attitudes, knowledge and behavior. |
| **Centrality**: how central the network's most central actor is in relation to how central all the other actors are ^5^ | While density and mean path describe the overall cohesion of a network, centralization describes the extent to which this cohesion is organized around particular actor(s) ^4^. High degree of centralization may have a positive effect on collective action as a central actor can prioritize certain action, and even coordinate the action, but it also indicates asymmetric influence relations ^4^. Hence, a highly centralized network would indicate a presence of central actors who are in key position to influence a large proportion of the landowner collective. |
| **Compartmentalization**: a presence of subgroups in which actors are joined together in tightly connected groups, between which there are fewer connections ^6^ | The low density of links between compartments may have negative effects on the collective action (see Density), and may lead to "them and us" attitudes, but high density inside compartments may lead to developing specialized knowledge ^4^. In the case of homophilic influence network, compartmentalization indicates subgroups of like-minded people with fewer connections (that would allow spread of attitudes and behavior) to people less similar to them. |
| **Bridging links:** links connecting different actor subgroups. Here, we measure bridging links as number of cut points (articulation points): actors whose removal increases the number of connected components in a network by forming two or more separate subgroups between which there are no connections ^3^. | By connecting network subgroups (potentially compartments), bridging links provide actors in subgroups access to external knowledge and resources. Thus, high number of bridges enable or support collective action among different groups of actors. In this study, we calculated number of cut-points (i.e. actors who connect subgroups/network components. Removal of cut-point actors leads to a network subgroup breaking into two with no communication in between) to indicate number of bridgers. |
| **Isolates**: non-connected actors | Actors who do not participate in local network because they do not have links to other actors (based on the network boundary setting). In our study, actors who had not had frequent, influential discussion about environmental performance of the farm. Our ‘Isolates’ network index shows number of isolates. |
| **Average weighted degree:** the average number of connections that actors in a network have, including the link weights. | Actor’s network degree is the number of immediate contacts that an actor has in a network, enabling access to knowledge, new ideas, resources, etc. In our study, link weight indicates influence. Thus, high average weighted degree would mean a network with strong peer influence among the actors. |

**Table S4. Structural social network indicators for actor similarity - based and** Erdős-Rényi (ER) **random networks**. The bold value is the mean (for which standard error (SD) is calculated), value above it the minimum value, and below it the maximum value for all simulations. Due to the high number of isolates in our networks, we have calculated some indices with and without isolates. Network size is the number of links in the network. Erdős–Rényi randomization was based on the average density of actor similarity - based networks, and should thus be similar for actor similarity - based and ER networks.

| SOCIAL NETWORK INDEX | ACTOR SIMILARITY - BASED  NETWORK | ACTOR SIMILARITY - BASED  SD | ER  NETWORK | ER NETWORK  SD |
| --- | --- | --- | --- | --- |
| Mean path length | 1.224  **2.210**  6.232 | 0.569 | 1.211  **2.528**  8.496 | 0.808 |
| Network size | 91.000  **142.726**  217.000 | 16.154 | 74.000  **139.117**  212.000 | 16.760 |
| Number of cut points | 26  **46.272**  70.000 | 6.175 | 8.000  **30.565**  60.000 | 6.522 |
| Centralization | 0.007  **0.015**  0.030 | 0.003 | 0.006  **0.016**  0.032 | 0.004 |
| Centralization  without isolates | 0.008  **0.021**  0.050 | 0.005 | 0.006  **0.025**  0.067 | 0.007 |
| Average weighted indegree | 0.289  **0.473**  0.729 | 0.057 | 0.205  **0.461**  0.704 | 0.060 |
| Average weighted indegree  without isolates | 0.523  **0.723**  0.979 | 0.060 | 0.642  **0.918**  1.225 | 0.068 |
| Compartmentalization | 0.210  **0.677**  0.911 | 0.095 | 0.791  **0.934**  0.974 | 0.019 |
| Density | 0.002  **0.004**  0.005 | 0.000 | 0.002  **0.003**  0.005 | 0.000 |
| Density without isolates | 0.006  **0.008**  0.012 | 0.001 | 0.010  **0.014**  0.021 | 0.001 |
| Isolates | 37.000  **69.262**  101.000 | 8.689 | 61.000  **99.657**  136.000 | 9.241 |

**Tables S5a-d.** Stepwise logistic regression analyses with forward selection were used to examine which variables could influence landowner’s probability to protect native habitat for biodiversity, and thus, be used as actor attributes. Tables S5a shows maximal models with variable “farm land contains native forest and shrubs and/or ungrazed tussock grassland” (Table S1 variable s1), and Table S5c maximal model with variable “All or part of the land is covenanted” (Table S1 variable s2) as dependent variables. S5b and S5c present the results, respectively. The results include only land use variables. This is partly because both our dependent variables were essentially describing land use. Further, it is likely that landowners’ values, ownership and choices required by the primary industry are already reflected in land use. Collinearity was detected only for land use variables. Significance codes for p-values: ***: < 0.001, ** < 0.01, * < 0.05, . < 0.1.

**Table S5a**.

| ID | Variable | Estimate | Std. Error | z value | Pr(>\|z\|) |  |
| --- | --- | --- | --- | --- | --- | --- |
|  | (Intercept) | -1.95E+00 | 2.11E+00 | -0.926 | 0.354311 |  |
| s7 | Private landowners should protect habitat for native plants and animals on private land: 7 | -5.31E-01 | 4.27E-01 | -1.241 | 0.214547 |  |
| s7 | Private landowners should protect habitat for native plants and animals on private land: 8 | -4.21E-01 | 4.23E-01 | -0.996 | 0.31948 |  |
| s7 | Private landowners should protect habitat for native plants and animals on private land: 9 | -7.86E-01 | 4.75E-01 | -1.655 | 0.097961 | . |
| s7 | Private landowners should protect habitat for native plants and animals on private land: 10 | -9.22E-01 | 4.80E-01 | -1.92 | 0.054872 | . |
| s8 | The Department of Conservation should protect habitat for native plants and animals on public land: 1 | -1.51E+01 | 3.96E+03 | -0.004 | 0.996959 |  |
| s8 | The Department of Conservation should protect habitat for native plants and animals on public land: 2 | -1.52E+01 | 1.94E+03 | -0.008 | 0.993766 |  |
| s8 | The Department of Conservation should protect habitat for native plants and animals on public land: 3 | -2.95E+01 | 1.95E+03 | -0.015 | 0.987893 |  |
| s8 | The Department of Conservation should protect habitat for native plants and animals on public land: 4 | -5.26E-01 | 2.37E+00 | -0.222 | 0.82414 |  |
| s8 | The Department of Conservation should protect habitat for native plants and animals on public land: 5 | 3.57E-01 | 2.11E+00 | 0.169 | 0.866028 |  |
| s8 | The Department of Conservation should protect habitat for native plants and animals on public land: 6 | -9.83E-01 | 2.07E+00 | -0.476 | 0.634054 |  |
| s8 | The Department of Conservation should protect habitat for native plants and animals on public land: 7 | -1.38E-01 | 2.04E+00 | -0.068 | 0.945994 |  |
| s8 | The Department of Conservation should protect habitat for native plants and animals on public land: 8 | -4.89E-01 | 2.03E+00 | -0.241 | 0.809854 |  |
| s8 | The Department of Conservation should protect habitat for native plants and animals on public land: 9 | 5.75E-02 | 2.04E+00 | 0.028 | 0.977491 |  |
| s8 | The Department of Conservation should protect habitat for native plants and animals on public land: 10 | 3.62E-01 | 2.03E+00 | 0.179 | 0.85801 |  |
| s2 | Has covenant on own land | 1.22E+00 | 3.30E-01 | 3.694 | 0.000221 | *** |
| s4 | Landownership: myself | -1.35E+00 | 1.14E+00 | -1.176 | 0.239723 |  |
| s4 | Landownership: another individual | -2.04E-01 | 4.18E-01 | -0.487 | 0.626112 |  |
| s4 | Landownership: family partnership | -1.66E-01 | 4.33E-01 | -0.384 | 0.701343 |  |
| s4 | Landownership: family trust | 2.64E+00 | 1.68E+00 | 1.568 | 0.116814 |  |
| s4 | Landownership: family company | -1.71E+01 | 1.14E+03 | -0.015 | 0.988042 |  |
| s4 | Landownership: māori trust / inc. | 3.98E-01 | 6.45E-01 | 0.618 | 0.536722 |  |
| s4 | Landownership: corporate owned | 6.35E-01 | 4.70E-01 | 1.35 | 0.177038 |  |
| s6 | Area | 8.61E-05 | 1.31E-04 | 0.658 | 0.510582 |  |
| s3 | Primary industry: grazing livestock, not own | -9.33E-01 | 6.90E-01 | -1.353 | 0.175908 |  |
| s3 | Primary industry: farming sheep | -4.61E-01 | 6.57E-01 | -0.701 | 0.483603 |  |
| s3 | Primary industry: farming beef | -5.64E-01 | 6.57E-01 | -0.857 | 0.391223 |  |
| s3 | Primary industry: dairying | 5.22E-01 | 1.25E+00 | 0.418 | 0.675597 |  |
| s3 | Primary industry: deer farming | 1.06E-01 | 1.12E+00 | 0.095 | 0.924602 |  |
| s3 | Primary industry: pig farming | -1.36E-01 | 1.54E+00 | -0.089 | 0.929438 |  |
| s3 | Primary industry: poultry farming | -1.49E+01 | 1.96E+03 | -0.008 | 0.993943 |  |
| s3 | Primary industry: other farmed livestock | -2.58E+00 | 1.42E+00 | -1.816 | 0.069396 | . |
| s3 | Primary industry: arable farming | -7.95E-02 | 1.07E+00 | -0.074 | 0.940855 |  |
| s3 | Primary industry: vegetable production | 3.43E-01 | 1.25E+00 | 0.275 | 0.782999 |  |
| s3 | Primary industry: kiwifruit production | -2.76E-01 | 2.54E+00 | -0.109 | 0.913228 |  |
| s3 | Primary industry: wine grape production | -3.23E+01 | 1.73E+03 | -0.019 | 0.985098 |  |
| s3 | Primary industry: growing fruits, nuts, edible tree crops | 3.94E-01 | 9.99E-01 | 0.394 | 0.693245 |  |
| s3 | Primary industry: exotic forestry | -1.39E+00 | 8.45E-01 | -1.642 | 0.100517 |  |
| s3 | Primary industry: farm-based tourism | 1.06E+00 | 1.25E+00 | 0.85 | 0.39556 |  |
| s3 | Primary industry: other | 1.56E+01 | 3.96E+03 | 0.004 | 0.996853 |  |
| s3 | Land use: cattle | 2.42E-01 | 2.95E-01 | 0.822 | 0.411148 |  |
| s1 | Land use: dairy platform | -1.03E+00 | 1.07E+00 | -0.962 | 0.33589 |  |
| s1 | Land use: dairy runoff | -1.64E-01 | 5.71E-01 | -0.287 | 0.773911 |  |
| s1 | Land use: deer | -5.51E-01 | 7.09E-01 | -0.777 | 0.437357 |  |
| s1 | Land use: flowers | -1.29E-01 | 1.27E+00 | -0.102 | 0.918701 |  |
| s1 | Land use: forestry | 2.31E+00 | 3.36E-01 | 6.879 | 6.05E-12 | *** |
| s1 | Land use: forestry harvested | 5.46E-01 | 8.39E-01 | 0.651 | 0.515249 |  |
| s1 | Land use: fruit | -6.23E-01 | 6.74E-01 | -0.925 | 0.354869 |  |
| s1 | Land use: grain seeds | -1.41E+00 | 6.86E-01 | -2.05 | 0.040335 | * |
| s1 | Land use: grapes | 1.67E+01 | 1.34E+03 | 0.012 | 0.990028 |  |
| s1 | Land use: grazing | 6.18E-02 | 3.73E-01 | 0.166 | 0.868158 |  |
| s1 | Land use: hay | 2.78E-01 | 3.00E-01 | 0.926 | 0.354459 |  |
| s1 | Land use: kiwifruit | -1.58E+00 | 2.20E+00 | -0.718 | 0.4726 |  |
| s1 | Land use: other livestock | 1.29E+00 | 5.55E-01 | 2.322 | 0.020216 | * |
| s1 | Land use: pigs | 9.09E-01 | 8.27E-01 | 1.1 | 0.27154 |  |
| s1 | Land use: poultry | -3.29E-01 | 9.04E-01 | -0.364 | 0.715963 |  |
| s1 | Land use: sheep and beef | 9.63E-01 | 3.77E-01 | 2.553 | 0.010682 | * |
| s1 | Land use: tourism | 1.31E-01 | 7.76E-01 | 0.168 | 0.866209 |  |
| s1 | Land use: vegetables indoors | -1.33E-01 | 1.20E+00 | -0.111 | 0.911427 |  |
| s1 | Land use: vegetables outdoors | 7.50E-01 | 5.97E-01 | 1.256 | 0.209152 |  |
| s1 | Land use: wetlands | 2.37E+00 | 3.79E-01 | 6.234 | 4.55E-10 | *** |
| s5 | Live on farm: 12985 | 7.31E-01 | 4.08E-01 | 1.79 | 0.073491 | . |
| s5 | Live on farm: 12986 | -1.75E-02 | 9.17E-01 | -0.019 | 0.984762 |  |
|  | Null deviance: 716.11 on 606 degrees of freedom  Residual deviance: 447.55 on 544 degrees of freedom  AIC: 573.55  Number of Fisher Scoring iterations: 16 | | | | | |

**Table S5b**.

| ID |  | Estimate | Std. Error | z value | Pr(>\|z\|) |  |
| --- | --- | --- | --- | --- | --- | --- |
|  | (Intercept) | -2.5721 | 0.2198 | -11.704 | < 2e-16 | ** |
| s2 | Has covenant on own land | 1.1942 | 0.2721 | 4.389 | 1.14e-05 | *** |
| s1 | Land use: forestry | 2.0378 | 0.2534 | 8.043 | 8.80e-16 | *** |
| s1 | Land use: sheep and beef | 0.8600 | 0.2351 | 3.659 | 0.000254 | *** |
| s1 | Land use: wetlands | 2.2560 | 0.3162 | 7.134 | 9.71e-13 | *** |
|  | Null deviance: 716.11 on 606 degrees of freedom  Residual deviance: 516.36 on 602 degrees of freedom  AIC: 526.36  Number of Fisher Scoring iterations: 5 | | | | | |

**Table S5c.**

|  |  | Estimate | Std. Error | z value | Pr(>\|z\|) |  |
| --- | --- | --- | --- | --- | --- | --- |
|  | (Intercept) | -17.63091 | 3733.47905 | -0.005 | 0.99623 |  |
| s7 | Private landowners should protect habitat for native plants and animals on private land: 7 | 0.89451 | 0.46726 | 1.914 | 0.05557 | . |
| s7 | Private landowners should protect habitat for native plants and animals on private land: 8 | 0.46092 | 0.4822 | 0.956 | 0.33913 |  |
| s7 | Private landowners should protect habitat for native plants and animals on private land: 9 | 0.72902 | 0.52367 | 1.392 | 0.16388 |  |
| s7 | Private landowners should protect habitat for native plants and animals on private land: 10 | 0.28499 | 0.54286 | 0.525 | 0.5996 |  |
| s8 | The Department of Conservation should protect habitat for native plants and animals on public land: 1 | -2.75869 | 7515.56255 | 0 | 0.99971 |  |
| s8 | The Department of Conservation should protect habitat for native plants and animals on public land: 2 | -2.34739 | 4704.04145 | 0 | 0.9996 |  |
| s8 | The Department of Conservation should protect habitat for native plants and animals on public land: 3 | -1.4432 | 4589.15314 | 0 | 0.99975 |  |
| s8 | The Department of Conservation should protect habitat for native plants and animals on public land: 4 | 15.94438 | 3733.47918 | 0.004 | 0.99659 |  |
| s8 | The Department of Conservation should protect habitat for native plants and animals on public land: 5 | 11.02069 | 3733.47939 | 0.003 | 0.99764 |  |
| s8 | The Department of Conservation should protect habitat for native plants and animals on public land: 6 | 14.45783 | 3733.47907 | 0.004 | 0.99691 |  |
| s8 | The Department of Conservation should protect habitat for native plants and animals on public land: 7 | 13.80726 | 3733.47907 | 0.004 | 0.99705 |  |
| s8 | The Department of Conservation should protect habitat for native plants and animals on public land: 8 | 14.07485 | 3733.47907 | 0.004 | 0.99699 |  |
| s8 | The Department of Conservation should protect habitat for native plants and animals on public land: 9 | 14.58785 | 3733.47907 | 0.004 | 0.99688 |  |
| s8 | The Department of Conservation should protect habitat for native plants and animals on public land: 10 | 14.65365 | 3733.47907 | 0.004 | 0.99687 |  |
| s4 | Landownership: myself | 2.05938 | 0.86788 | 2.373 | 0.01765 | * |
| s4 | Landownership: another individual | 0.73929 | 0.49602 | 1.49 | 0.1361 |  |
| s4 | Landownership: family partnership | 1.14743 | 0.5035 | 2.279 | 0.02267 | * |
| s4 | Landownership: family trust | 2.84305 | 1.60545 | 1.771 | 0.07658 | . |
| s4 | Landownership: family company | -15.16504 | 2181.20979 | -0.007 | 0.99445 |  |
| s4 | Landownership: māori trust / inc. | -0.32782 | 0.89255 | -0.367 | 0.7134 |  |
| s4 | Landownership: corporate owned | 0.80028 | 0.56051 | 1.428 | 0.15335 |  |
| s3 | Primary industry: grazing livestock, not own | -1.00488 | 0.70147 | -1.433 | 0.15199 |  |
| s3 | Primary industry: farming sheep | -1.1933 | 0.69733 | -1.711 | 0.08704 | . |
| s3 | Primary industry: farming beef | -0.63404 | 0.67011 | -0.946 | 0.34405 |  |
| s3 | Primary industry: dairying | -0.4815 | 1.32175 | -0.364 | 0.71564 |  |
| s3 | Primary industry: deer farming | -1.41502 | 1.65951 | -0.853 | 0.39384 |  |
| s3 | Primary industry: pig farming | -1.62701 | 1.72871 | -0.941 | 0.34662 |  |
| s3 | Primary industry: poultry farming | -15.77646 | 3237.32355 | -0.005 | 0.99611 |  |
| s3 | Primary industry: other farmed livestock | 0.18893 | 1.12645 | 0.168 | 0.8668 |  |
| s3 | Primary industry: arable farming | -0.6377 | 0.97659 | -0.653 | 0.51376 |  |
| s3 | Primary industry: vegetable production | -17.60586 | 1684.43689 | -0.01 | 0.99166 |  |
| s3 | Primary industry: kiwifruit production | -21.86036 | 1252.39174 | -0.017 | 0.98607 |  |
| s3 | Primary industry: wine grape production | 0.31805 | 3547.36647 | 0 | 0.99993 |  |
| s3 | Primary industry: growing fruits, nuts, edible tree crops | -2.61233 | 1.22786 | -2.128 | 0.03337 | * |
| s3 | Primary industry: exotic forestry | -1.43336 | 1.10572 | -1.296 | 0.19487 |  |
| s3 | Primary industry: farm-based tourism | -0.30471 | 1.22571 | -0.249 | 0.80367 |  |
| s3 | Primary industry: other | 17.84228 | 6522.63866 | 0.003 | 0.99782 |  |
| s1 | Land use: bush | 1.33585 | 0.31871 | 4.191 | 2.77E-05 | *** |
| s1 | Land use: cattle | 0.25264 | 0.32223 | 0.784 | 0.43303 |  |
| s1 | Land use: dairy platform | 0.04347 | 1.16905 | 0.037 | 0.97034 |  |
| s1 | Land use: dairy runoff | 0.56677 | 0.51588 | 1.099 | 0.27192 |  |
| s1 | Land use: deer | -1.47072 | 0.90462 | -1.626 | 0.10399 |  |
| s1 | Land use: flowers | -16.51415 | 2108.4609 | -0.008 | 0.99375 |  |
| s1 | Land use: forestry | 0.07809 | 0.34919 | 0.224 | 0.82305 |  |
| s1 | Land use: forestry harvested | -0.40126 | 1.0045 | -0.399 | 0.68956 |  |
| s1 | Land use: fruit | 0.18262 | 0.72978 | 0.25 | 0.8024 |  |
| s1 | Land use: grain seeds | 0.13595 | 0.58743 | 0.231 | 0.81699 |  |
| s1 | Land use: grapes | -17.14267 | 3038.8154 | -0.006 | 0.9955 |  |
| s1 | Land use: grazing | 0.09179 | 0.38977 | 0.235 | 0.81383 |  |
| s1 | Land use: hay | -0.26423 | 0.32098 | -0.823 | 0.41039 |  |
| s1 | Land use: kiwifruit | 4.42176 | 1.36611 | 3.237 | 0.00121 | ** |
| s1 | Land use: other livestock | -0.26251 | 0.6334 | -0.414 | 0.67855 |  |
| s1 | Land use: pigs | 0.75615 | 0.82544 | 0.916 | 0.35964 |  |
| s1 | Land use: poultry | -1.45112 | 1.32678 | -1.094 | 0.27408 |  |
| s1 | Land use: snb | 0.81476 | 0.41607 | 1.958 | 0.0502 | . |
| s1 | Land use: tourism | 0.60351 | 0.86389 | 0.699 | 0.48481 |  |
| s1 | Land use: vegetables indoors | -16.80093 | 1827.911 | -0.009 | 0.99267 |  |
| s1 | Land use: vegetables outdoors | -0.16404 | 0.70244 | -0.234 | 0.81535 |  |
| s1 | Land use: wetlands | 1.02729 | 0.36046 | 2.85 | 0.00437 | ** |
| s5 | Live on farm: 12985 | -0.22256 | 0.46764 | -0.476 | 0.63412 |  |
| s5 | Live on farm: 12986 | 1.5973 | 0.97573 | 1.637 | 0.10162 |  |
|  | Null deviance: 559.15 on 606 degrees of freedom  Residual deviance: 396.01 on 545 degrees of freedom  AIC: 520.01  Number of Fisher Scoring iterations: 17 | | | | | |

**Table S5d.**

|  |  | Estimate | Std. Error | z value | Pr(>\|z\|) |  |
| --- | --- | --- | --- | --- | --- | --- |
|  | (Intercept) | -2.8859 | 0.2793 | -10.335 | < 2e-16 | *** |
| s1 | Land use: bush | 1.2523 | 0.2513 | 4.983 | 6.27e-07 | *** |
| s1 | Land use: dairy platform | 0.8408 | 0.3217 | 2.614 | 0.008960 | ** |
| s1 | Land use: sheep and beef | 0.7732 | 0.2875 | 2.689 | 0.007165 | ** |
| s1 | Land use: wetlands | 0.9950 | 0.2937 | 3.388 | 0.000703 | *** |
|  | Null deviance: 559.15 on 606 degrees of freedom  Residual deviance 484.38 on 596 degrees of freedom  AIC: 494.38  Number of Fisher Scoring iterations: 5 | | | | | |

# APPENDIX A. OVERVIEW, DESIGN CONCEPTS AND DETAILS PROTOCOL FOR LANDOWNER MODEL.

The following model description follows the ODD (Overview, Design concepts, Details) protocol for agent-based models ^7,8^.

A1.1. Purpose

We designed and implemented the model to simulate collectively achieved conservation action on agricultural land, and to quantify the environmental outcomes of landowners’ individual action with spatial landscape indicators (area and fragmentation of protected land). Specifically, the model allows us to investigate:

1) how the interacting effects of peer network influence, cross-scale actor influence, actor attributes and spatial knowledge diffusion influence landscape-level conservation of native forest on agricultural land.

2) the mechanisms through which multiple social influences affecting social actor’s decision-making translates into changes in landscape structure. I.e., which social influences, or combination of the social influences included, frequently produce desirable environmental outcomes.

## A1.2. Entities, state variables, scales

The model includes four types of entities:

1. Agents/individuals: landowners are social actors who make decisions about the protection of native or semi-natural habitat on their land. They are characterized by actor attributes (Table S1) and make decisions on whether their land (“farm”) is protected or not.
2. Agents/individuals: cross-scale actors are social actors who influence landowners connected to them. Cross-scale actors include representatives from indigenous groups (iwi), regional and district councils and central government. Cross-scale actors’ status does not get updated; their influence is always pro-conservation and affects directly only those landowners, who have (influential) links with cross-scale actors.
3. Spatial units: farms consist of multiple patches (grid cells) and represent area that each landowner has available for conservation, and upon which landownermakes decisions. A farm can be in three states: protected, unprotected or covenanted.
4. Spatial units: all patches are additionally considered ecological spatial units, upon which natural habitat area and fragmentation indices are calculated. Connected protected patches are considered to create a non-fragmented habitat area. On the contrary, any non-protected cells between protected patches indicates the presence of habitat edges.

The variables characterizing these entities are presented in Table S1.

There are 200 social actors in the model, who are all landowners. Cross-scale actors are included as cross-scale social network variables of landowners. The number of grid cells in the model arena is 841. Farms represent the land area that each landowner could set as protected natural habitat, and their sizes are standardized to give each landowner land proportionally to the size of their largest land parcel (in case a survey responder’s farm consists of multiple blocks of land), self-reported in the survey. The model was run for 150 time steps, which represents 15 years of time.

## A1.3. Process overview and scheduling

*General model concept* *and simulations*

The model is an agent-based model ^9^ integrated with the R environment ^10^, based on a new and detailed dataset on 600 rural decision-makers ^1^. The model consists of i) landowners and their social networks (two-mode network, i.e. including connectivity between landowners, and between landowners and cross-scale actors), and ii) a simulated landscape consisting of two layers: farms upon which each landowner makes decision to protect, covenant or not protect land, and an ecological landscape representing land as natural habitat or land in other use.

We ran one simulation for each parameter value combination for the two experiments including all social influence types (Multiple Influences experiments), which resulted in 6561 simulations for actor similarity - based and Erdős-Rényi (ER) network each. Since the number of unique parameter value combinations were lower for the two other experiments including only social network influence (from landowners, i.e. excluding cross-scale actors) and actor attributes, we ran 80 simulations for each combination to gain 6561 simulations for the actor similarity - based and ER network each. The model adjusts the assigned social influence and actor attribute weights (presented in manuscript Table 1) to sum to one, and the simulations and result analyses are performed on the adjusted values.

For each simulation (Figure S3), a baseline system is set by drawing a subset of 200 individuals from a data set of 600 landowners, with their individual, self-reported actor attributes. A landscape is constructed with actor attribute data on ownership of natural habitats, covenanted land and farm size. During each time step, probabilities for protecting natural habitat are calculated for a subset (the percentage of landowners chosen for decision-making is included in parameter value settings, see Table 1) of landowners, who then either protect or not protect their land. For those not chosen for decision-making, we assume a decision to continue with their current behavior. If a landowner decides to protect their land, the farm is marked as protected area. If a landowner decides to stop protecting land, the land is marked as non-protected. Those landowners who have decided to protect land also have an option to covenant land, in which case that land cannot become unprotected during the entire simulation. During the following time step, decisions are made in an updated social-ecological context, since the landscape may have changed as may have the behavioral status of network actors. The model stops after 150 time steps. Model output (state) variables (Table S2) capture changes at the landscape-level in protected area extent, fragmentation (count of fragments, entropy) and the duration of land as protected area, as well as number of landowners participating in conservation, and the detailed network structure of landowners’ network.

###
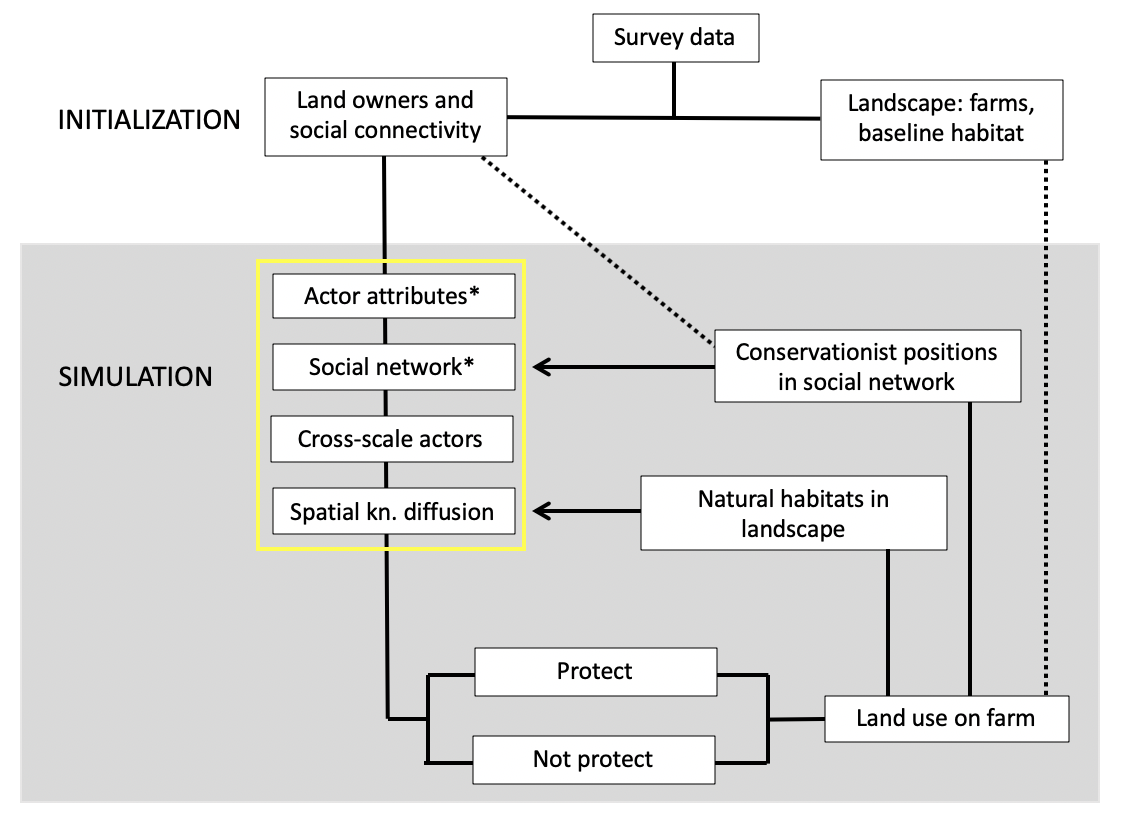


**Figure S3. Model logic.** Survey data is used in initialization to create landowners (including actor attributes), their network connections, and initial landscape. On the first time step of a simulation, social network influence and spatial knowledge diffusion (“spatial kn. diffusion”) in decision-making are based on survey data; indicated with dashed lines. Yellow box marks decision-making based on social influence and landowners’ individual actor attributes. Based on these influences, landowners decide to either protect or not protect land on their farm, potentially changing land use (i.e. protected/not) on farm. Those who protect land are called conservationists in the figure. The extent of natural habitats on landscape-level emerges from land use on farms, and affects spatial knowledge diffusion in decision-making during the next time step. Another social-ecological feedback is formed by social network influence, which is updated on each time step, as land use on farm determines which landowners in the network have protected natural habitats (here shortened to “conservationists”). Asterisks (*) mark the influences that are included in decision-making in Ingroup Influence experiments.

A1.4. NetLogo pseudocode

INITIALIZATION

**Create landowner networks and calculate social network indices**

Read in an input file with actor attributes for 600^[[1]](#footnote-1)^ survey respondents/landowners.

Create landowner agents by drawing a random subsample of landowners from actor attribute input file.

Define survey data actor attributes as landowner variables (see Table S1).

If ER Network generation is not chosen:

load actor similarity matrix from R environment

extract data for landowners from actor similarity matrix based on their ID

add network link between those landowners that are connected in actor similarity

matrix, according to their self-reported indegree (e.g. number of incoming links)

set self-reported influence from other actors (converted to quantitative values: [0.33 0.66 1], non- influential links have value 0 and are not included) as link

weights

If actor similarity – based networkis not chosen:

ask landowners to create network links with other landowners in given probability

using ER network generation

set [0.33 0.66 1] as link weight list

set network link weights randomly from link weight list

Export a list of IDs for landowners (i.e. landowner network actors)

Export weighted matrix of landowners network.

Social network analysis is performed in R environment, using mainly iGraph package ^11^.

Import network indices from R environment to NetLogo.

**Create farms and landscape**

Create as many farms as there are landowners.

Use survey data on normalized farm areas of the landowners to set the size of each landowner's farm. Set landowner ID as farm ID.

Cluster together all patches that belong to the same farm.

Mark adjacent farms to each farm as its ‘neighbor farms’.

Fill landscape: while patches without farm ID, continue adding edge patches to farms.

Set patch colour the colour of the farm.

Set the colour of the patch as ‘start patch colour’.

**Set baseline native habitants (i.e. protected land)**

Landowners with native habitat on their land (survey data):

mark own farm as protected land.

Landowners with native forest on their land who have joined a conservation covenant (survey data):

mark own farm as protected land, covenanted land, and as inconvertible.

Report baseline count of patches that contain native forest.

Report baseline count of patches that are part of covenant.

All other patches:

set patches as not protected, not inconvertible, and not baseline habitat.

**Decision-making set-up**

Set values for ‘landowner weight’ (ingroup influence), ‘actor attributes weight’, ‘indigenous weight’, ‘council weight’, ‘central government weight’ and ‘spatial knowledge diffusion weight’ (the weights that each behavioral driver has on decision-making, see manuscript Table 1) according to the experiment-specific parameter values.

Adjust the total value of ‘landowner weight’, ‘actor attributes weight’, ‘indigenous weight’, ‘council weight’, ‘central gov weight’ and ‘spatial knowledge diffusion weight’ to sum to one. Set parameter values for number Change-makers, i.e. the percentage of landowners able to change the conservation status of their farms on each time step, as well as ‘Time steps’, i.e. the number of ticks that landowners have to wait until next change of conservation status on their land.

EXECUTION

To go:

If time < stop time:

**Update**

Set patch colour ‘start patch colour’

Set ‘decision-makers’ those landowners for whom ‘minimum time since change’ has passed or who have not changed the conservation status of their farm yet.

**Calculate landowner (ingroup) network influence**

Landowners with indegree > 0:

let my ‘habitat connections’ be landowners that I have incoming link with and who have native habitat on their farms

calculate the fraction of habitat connections of all my incoming links, both multiplied with link weights.

set the fraction as my ‘network influence’

Landowners with indegree = 0:

set my ‘landowner network influence’ to 0

**Calculate cross-scale influence**

Landowners:

set my ‘indigenous group influence’ my number of links to indigenous group multiplied with their link weight

set my ‘council group influence’ my number of links to council representatives multiplied with their link weight

set my ‘central government group influence’ my number of links to central

government representatives multiplied with their link weight

Set the highest indigenous/council/central government group influences among landowners as maximum indigenous/council/central government group influence among the landowners.

Landowners with indigenous group degree > 0 and indigenous representatives link weight > 0

set indigenous representatives influence as my indigenous group influence divided with maximum indigenous group influence

Landowners with indigenous group degree = 0 or indigenous group link weight = 0

set indigenous influence 0

Calculate council and central government group influences for each landowner similarly to indigenous group influence.

**Calculate actor attribute influence**

Landowners:

set my actor attributes influence for protecting land and for covenanting land based on regression model (manuscript equation 4).

**Calculate spatial knowledge diffusion influence**

Landowners:

set ‘my spatial knowledge influence’ as a count of my neighbour farms with protected land divided with the total count of my neighbour farms

**Make conservation decisions**

‘Decision-makers’:

set my ‘conservation probability’ as weighted sum of behavioral factors and their parameter values (manuscript equation 5).

**Implement conservation action**

If my ‘conservation probability’ is > random floating-point number 0-1

PROTECT NATIVE HABITAT

if my farm is not inconvertible

mark as protected land

change patch colour to white

‘Decision-makers’ without covenant barriers:

if my ‘covenant attributes’ > random floating-point number 0-1

if my farm is marked as protected land and is

not inconvertible

JOIN COVENANT

mark land as inconvertible

set patch colour blue

Else:

UNPROTECT NATIVE HABITAT

If my farm is not inconvertible

mark as unprotected land

change patch colour to black

**Update the conservation status of landowners and land**

If land marked as protected

set landowner as ‘conservationist’

Else:

set landowner as ‘non-conservationist’

If land marked as covenant

set landowner as ‘covenant landowner’

Else:

set landowner as ‘non- covenant landowner’

Report the number of conservationists

Report the number of covenant landowners

**Calculate the residence time of each farm as protected land** *(not used in this study)*

If patch colour white:

set ‘residence time’ as (‘residence time’ + 1)

If patch colour blue:

set ‘residence time’ as (‘residence time’ + 1)

**Calculate habitat fragmentation indices** (Modified from ^12^)

**Identify contiguous groupings of patches with protected land as fragments** (Modified from ^12^)

Mark patches that are not protected land to a group of its own.

Other patches: (loop)

select one protected land patch as a start of a new fragment

if a neighbouring patch is marked as protected land, assign it to the same fragment as a starter patch and mark as assessed

if a neighbouring patch is marked as protected land, assign it to the same fragment as a starter patch and mark as assessed

continue until all patches with protected land have been assigned into fragments

**Calculate number and size of habitat fragments** (Modified from ^12^)

Mark patches that are not protected land as a group of its own.

Label fragments with identification numbers

With each new fragment ID number, set ‘fragment count’ (fragment count + 1)

Set number of patches in each fragment as their area

Report fragment count and minimum and maximum fragment areas

Tick

If time = stop time:

**Export habitat coordinates and social network data**

Export a list of coordinates for each patch with protected land to R environment for calculating entropy

Export a list of IDs and conservationist status of landowners (social network actors) and weighted matrix of landowners network (used for creating manuscript Figure S2 graph).

**Calculate environmental outcome variables**

Import entropy value from R environment

Report outcome variables, listed in Tables S2 and S4.

## A1.5. Design concepts

**A1.5.1. Basic principles**

General concepts and hypotheses, and their relationship to the model:

- Biodiversity is affected by the abundance and spatial configuration of habitats on landscape level ^13^. The model investigates landowners’ collective capacity to protect natural habitats on landscape-level and quantifies the environmental results with indicators describing the area and fragmentation of natural habitats on a landscape.
- Emergence ^14^: landscape-level area and connectivity of natural habitats in agricultural land is produced collectively by land use on farms, which, in turn, is a result of individual decision-making of landowners.
- Environmental outcomes of collective action may range from highly ineffective to successful ^15^. The output variables enable capturing the success of collective action.
- Pro-environmental behavior can be encouraged by social setting ^16^. Two to four social influence types are included in the study experiments.
- Multiple social influences’ effects are not necessarily additive, and the interplay between social influence types and constraints may influence environmental outcomes emerging from individual landowners’ behavior ^15,17^. The model allows including several behavioral factors in decision-making and investigating the effects that the factors have in diverse combinations.

**A1.5.2. Emergence**

In the model, the macro-level environmental outcome (landscape structure) emerges from micro-level social interactions, i.e. individual decision-making and consequent farm-level land use and social network influence. The micro-level interactions are, in turn, affected by the macro-level environmental change (spatial knowledge diffusion) and social diffusion (social network influence).

**A1.5.3. Adaptation and objectives**

The model includes heterogeneity in the social component, which allows landowners to have diverse objectives. Adaptation in a narrow sense is present in that landowners’ decision-making takes into account changes in neighbouring farms. However, while landowners respond to changing conditions, seeking a specific individual or collective goal in their action is not included in the model.

**A1.5.4. Learning**

Not included

**A1.5.5. Prediction**

Not included

**A1.5.6. Sensing**

The external variables included in landowners’ decision-making are spatial knowledge diffusion, which is theoretically based on landowners observing conservation action on neighbouring farms.

**A1.5.7. Interaction**

Landowners interact directly through static network links, and indirectly through social network and spatial knowledge diffusion, when in effect.

**A1.5.8. Stochasticity**

Stochasticity is included partly to protect the identity of survey repliers, and partly to account for unknown factors in decision-making. The following processes are randomized:

- the location of each farm on the landscape

- the sample of 200 landowners from a pool of 600 surveyed landowners

- randomized network: an Erdös-Rényi randomized network model

- actor similarity - based network construction: which pairs of landowners are connected is partly random (due to unique samples of landowners for each simulation) and based on linking landowners who are alike each other in actor attributes. See manuscript Methods and Materials section.

**A1.5.9. Collectives**

The social community of the model is conceptually a two-level network, in which landowners produce a layer one where they have links with each other as well as to the cross-scale actors. On the cross-scale actors' layer, links between cross-scale actors are not constructed, they have links only to the landowners.

**A1.5.10. Observation**

Data collected from model simulations is listed in Table S2.

State variables that are kept constant are:

- number of landowners

- actor attributes

- networks connections and link weights (networks are static during a simulation run, but positions in the network occupied by those who have protected land change according to landowner actions)

- time (number of ticks) until the model stops, i.e. each simulation runs for 150 time steps.

## A1.5.11. Initialization

For each simulation, a subset of 200 individuals is drawn from a dataset of 600 landowners, with their individual, self-reported actor attributes. Landowners’ network is created based on actor similarity matrix and actor attribute data, or ER randomization. A landscape is constructed with actor attribute data on ownership of natural habitats, covenanted land and farm sizes. Initialization is described in detail in Pseudocode: initialization section.

## A1.5.12. Input data

- Actor attributes: a table that includes 600 landowners and their actor attributes. Table S5 provides a sample of the table. The entire input file is available from Brown, P. on reasonable request.

- Farm sizes: standardized (0 – 1) farm area of every landowner (Table S5)

- Actor similarity matrix (Table S6): a matrix that links landowners to each other according to their similarity, based on their actor attributes data. The values in the matrix are link weights that represent self-reported level of influence between landowners, and degree is self-reported in survey data.

## A1.5.13. Submodels

The model’s procedures and sub-models and summarized in Materials and Methods section in manuscript, Supplementary Materials Figure S3 and Appendix A section A1.4.: Pseudocode.

# A2. MODEL EVALUATION

Parameter sensitivity analysis for the model is essentially performed as the main analysis of the study. The main results are presented in manuscript Figure 3. Suitability of the model for its objectives and validity of input data and model outputs are discussed in the manuscript in Discussion section (uncertainty and caveats).

TABLES S5-S7 (MODEL INPUT FILES) ARE AVAILABLE AS SEPARATE FILES.

**Table S6. Model input data for actor attributes.** A sample for 100 landowners.

**Table S7. Actor similarity matrix.** Example of actor similarity matrix that is used as an input data

**Table S8.** **Input data for calculating Gower’s similarity.** Actor attributes matrix used to create actor similarity - based networks.

SUPPLEMENTARY REFERENCES

1. Brown, P. & Roper, S. Innovation and networks in New Zealand farming. *Aust. J. Agric. Resour. Econ.* 61, 422–442 (2017).

2. Shannon, C. E. A Mathematical Theory of Communication. *Bell Syst. Tech. J.* **27**, 379–423 (1948).

3. Prell, C. *Social network analysis*. (SAGE publications Ltd, 2012).

4. Bodin, Ö. & Crona, B. I. The role of social networks in natural resource governance: What relational patterns make a difference? *Glob. Environ. Chang.* **19**, 366–374 (2009).

5. Freeman, L. C. Centrality in social networks conceptual clarification. *Soc. Networks* **1**, 215–239 (1978).

6. Girvan, M. & Newman, M. E. J. Community structure in social and biological networks. *Proc. Natl. Acad. Sci. U. S. A.* **99**, 7821–7826 (2002).

7. Grimm, V. *et al.* A standard protocol for describing individual-based and agent-based models. *Ecol. Modell.* **198**, 115–126 (2006).

8. Grimm, V. *et al.* The ODD protocol: A review and first update. *Ecol. Modell.* **221**, 2760–2768 (2010).

9. Wilensky, U. NetLogo. (1999).  http://ccl.northwestern.edu/netlogo/. Center for

Connected Learning and Computer-Based Modeling, Northwestern University,

Evanston, IL.

10. R Core Team. R: A language and environment for statistical computing. R Foundation for Statistical Computing, Vienna, Austria. URL https://www.R-project.org/. (2018).

11. Csardi, G. & Nepusz, T. The igraph software package for complex network research, Interjournal, Complex Systems 1695. http://igraph.org. (2006).

12. Martin, A. E. & Fahrig, L. Reconciling contradictory relationships between mobility and extinction risk in human‐altered landscapes. *Funct. Ecol.* **30**, 1558–1567 (2016).

13. Hanski, I. Habitat loss, the dynamics of biodiversity, and a perspective on conservation. *Ambio* **40**, 248–255 (2011).

14. Levin, S. A. Self-organization and the Emergence of Complexity in Ecological Systems. *Bioscience* **55**(12), 1075 -1079 (2005).

15. Bodin, Ö. Collaborative environmental governance: Achieving collective action in social-ecological systems. *Science* **357(3652)**, eaan1114 (2017).

16. Cinner, J. E. How behavioral science can help conservation. *Science.* **362(6417)**, 889–891 (2018).

17. Côté, I. M., Darling, E. S. & Brown, C. J. Interactions among ecosystem stressors and their importance in conservation. *Proc. R. Soc. B* **283**, 20142592 (2016).

1. A subset of 3311 survey respondents. The subset of 600 landowners is a result of eliminating "Non-Applicable" - answers (mainly due to survey logic, occassionally because a responder has chosen to not reply to a specific question) from survey response variables used in the regression analyses. [↑](#footnote-ref-1)
